# Comparative Genomics of Completely Sequenced *Lactobacillus helveticus* Genomes Provides Insights into Strain-Specific Genes and Resolves Metagenomics Data Down to the Strain Level

**DOI:** 10.3389/fmicb.2018.00063

**Published:** 2018-01-30

**Authors:** Michael Schmid, Jonathan Muri, Damianos Melidis, Adithi R. Varadarajan, Vincent Somerville, Adrian Wicki, Aline Moser, Marc Bourqui, Claudia Wenzel, Elisabeth Eugster-Meier, Juerg E. Frey, Stefan Irmler, Christian H. Ahrens

**Affiliations:** ^1^Agroscope, Research Group Molecular Diagnostics, Genomics and Bioinformatics, Wädenswil, Switzerland; ^2^Swiss Institute of Bioinformatics, Wädenswil, Switzerland; ^3^Agroscope, Research Group Biochemistry of Milk and Microorganisms, Bern, Switzerland; ^4^School of Agricultural, Forest and Food Sciences HAFL, Bern University of Applied Sciences, Zollikofen, Switzerland

**Keywords:** whole genome sequencing, PacBio, comparative genomics, strain-specific genes, CRISPR/Cas, metagenomics, natural whey starter cultures, dairy industry

## Abstract

Although complete genome sequences hold particular value for an accurate description of core genomes, the identification of strain-specific genes, and as the optimal basis for functional genomics studies, they are still largely underrepresented in public repositories. Based on an assessment of the genome assembly complexity for all lactobacilli, we used Pacific Biosciences' long read technology to sequence and *de novo* assemble the genomes of three *Lactobacillus helveticus* starter strains, raising the number of completely sequenced strains to 12. The first comparative genomics study for *L. helveticus*—to our knowledge—identified a core genome of 988 genes and sets of unique, strain-specific genes ranging from about 30 to more than 200 genes. Importantly, the comparison of MiSeq- and PacBio-based assemblies uncovered that not only accessory but also core genes can be missed in incomplete genome assemblies based on short reads. Analysis of the three genomes revealed that a large number of pseudogenes were enriched for functional Gene Ontology categories such as amino acid transmembrane transport and carbohydrate metabolism, which is in line with a reductive genome evolution in the rich natural habitat of *L. helveticus*. Notably, the functional Clusters of Orthologous Groups of proteins categories “cell wall/membrane biogenesis” and “defense mechanisms” were found to be enriched among the strain-specific genes. A genome mining effort uncovered examples where an experimentally observed phenotype could be linked to the underlying genotype, such as for cell envelope proteinase PrtH3 of strain FAM8627. Another possible link identified for peptidoglycan hydrolases will require further experiments. Of note, strain FAM22155 did not harbor a CRISPR/Cas system; its loss was also observed in other *L. helveticus* strains and lactobacillus species, thus questioning the value of the CRISPR/Cas system for diagnostic purposes. Importantly, the complete genome sequences proved to be very useful for the analysis of natural whey starter cultures with metagenomics, as a larger percentage of the sequenced reads of these complex mixtures could be unambiguously assigned down to the strain level.

## Introduction

Lactic acid bacteria (LAB) degrade sugar to lactic acid and are often used in food fermentation (Leroy and De Vuyst, 2004; Giraffa et al., 2010). *Lactobacillus* is one of several genera that belong to LAB (Sun et al., 2015) and, due to their role in fermented food production or their use as probiotics, they are among the most important bacteria in food microbiology (Salvetti et al., 2012). In general, lactobacilli are microaerophilic, Gram-positive bacteria that form rods or cocci (Makarova et al., 2006). In milk, lactobacilli degrade lactose, citric acid, milk proteins, and lipids (McSweeney, 2011). The breakdown of milk proteins is considered to contribute the most to the development of flavor. In addition, various metabolites formed by these biochemical activities are precursors for aroma-active compounds.

*Lactobacillus helveticus* strains are abundant in the natural whey starter cultures (NWCs) that are used for the production of Gruyère, a protected designation of origin (PDO) cheese (Moser et al., 2017) (http://gruyere.com/en/specifications/?vt=ch). *L. helveticus* exhibits diverse proteolytic and peptidolytic activities. It is widely used with other thermophilic LAB, including *Streptococcus thermophilus* and *Lactobacillus delbrueckii* subsp*. lactis*, in the manufacture of Swiss cheese and Italian hard cheeses (Slattery et al., 2010; Eugster-Meier et al., 2017) such as Grana Padano, Parmigiano Reggiano, and Provolone, and as flavor-enhancing adjunct culture in Cheddar cheesemaking (Hannon et al., 2007; Slattery et al., 2010). Several *L. helveticus* strains may be exploited as probiotics that provide health-promoting properties (Taverniti and Guglielmetti, 2012).

The computational mining of genome sequences, for example for genes encoding specific metabolic activities, or involved in toxin formation, may facilitate the selection of strains for specific biotechnological applications. Next-generation sequencing (NGS) technologies such as the cost-efficient Illumina short read sequencing technology have been widely used to sequence bacterial genomes (Mavromatis et al., 2012). However, the presence of repeated sequences such as insertion sequence (IS) elements and rDNA operons severely compromises the ability to completely assemble complex genomes (Koren et al., 2013). Accordingly, although the number of publicly available bacterial genome assemblies has been increasing exponentially (Reddy et al., 2014), the large majority has been reported as draft genomes with a large number of contigs, which represents a serious limitation for follow-up analyses (Ricker et al., 2012). This is particularly relevant for LAB which often harbor many repeats and IS elements (Cahill et al., 2010; Sun et al., 2015). The phylogeny of LAB has recently been resolved in fine detail (Sun et al., 2015). However, only two *L. helveticus* strains were included in that study and almost all genome assemblies were fragmented, prompting the authors to emphasize the need to add more complete genome sequences in the future. The value of complete genome sequences, both to accurately describe the pan-core genome and to identify functions uniquely encoded in individual strains, is obvious, as is the value of the development of specific diagnostic tests (Ercolini, 2013; Hornischer and Häußler, 2016) or to study genome re-arrangements, adaptation, and evolution (Ricker et al., 2012).

The first complete *L. helveticus* genome sequence was that of strain DPC 4571, a cheese isolate. The shotgun-sequenced genome harbored a remarkably high number of IS elements (213) and 141 non-transposase encoding pseudogenes (Callanan et al., 2008). In 2013, the complete genome of strain CNRZ 32, a strain used as a commercial cheese flavor adjunct and for the production of bioactive peptides in milk, was published (Broadbent et al., 2013). Similar to strain DPC 4571, CNRZ 32 harbored a large number of repeats (356 IS elements and 163 non-transposase encoding pseudogenes). The unusually high number of IS elements and pseudogenes indicates ongoing genome degeneration (Callanan et al., 2008; Broadbent et al., 2013), a feature that has also been observed in other dairy species such as *Lactobacillus casei* (Cai et al., 2009), *S. thermophilus* (Bolotin et al., 2004), and *L. delbrueckii* (Makarova et al., 2006). A comparison of five *L. helveticus* strains sequenced with second-generation short read NGS technologies (Illumina or Roche 454) was published in 2013 (Cremonesi et al., 2013).

To circumvent the problems associated with short reads, we used long reads from Pacific Biosciences' (PacBio) third-generation NGS technology (Eid et al., 2009) and state-of-the-art assembly algorithms (Koren et al., 2012; Chin et al., 2013) to sequence and *de novo* assemble the complete genomes of three *L. helveticus* isolates from the dairy environment. The analysis of the repeat structure for all LAB indicated that PacBio long reads should be particularly suitable to *de novo* assemble genomes that contain a large number of repetitive sequences or IS elements. Here, we present the results of our study providing a phylogenetic profile of *Lactobacillales* with a focus on *L. helveticus*, and the first pan-core genome analysis based on 12 completely sequenced *L. helveticus* strains. Notably, the complete genome sequences proved to be very useful for analyzing NWCs with metagenomics, as a larger percentage of the sequenced reads of these complex mixtures could be unambiguously assigned even down to the strain level.

## Materials and methods

### Repeat analysis

**Figure 1:**
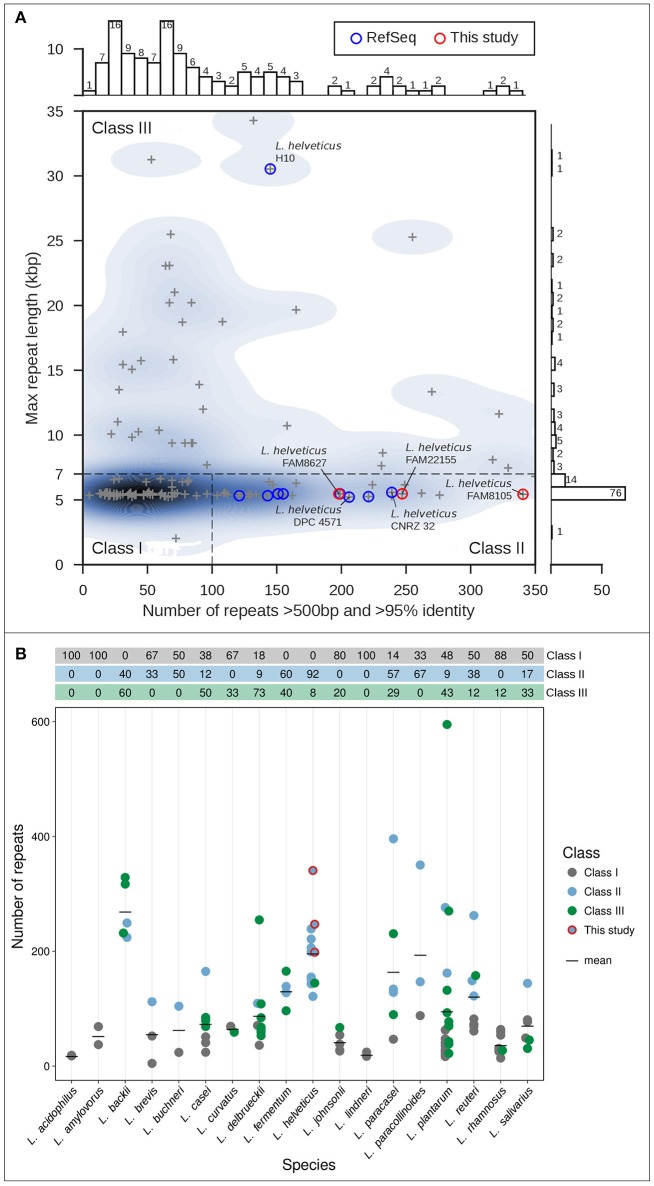
Classification of genome assembly difficulty (Koren et al., 2013) for selected completely sequenced genomes. **(A)** Genome assembly difficulty for genomes of the genus *Lactobacillus* including nine completely sequenced *L. helveticus* genomes (blue circles) and the three FAM strains sequenced in this study (red circles). For each genome, the length of the longest repeat (in kbp; y-axis) is plotted vs. the number of repeats (greater 500 bp), with more than 95% sequence identity (x-axis). The three classes of genome assembly difficulty are shown (roman numerals). **(B)** Overview of the total number of repeats for different *Lactobacillus* strains grouped according to species. Species with only one strain were excluded from the analysis. The colors indicate the assembly difficulty classification; the table on top of the graph shows the percentages per class indicated by color. The black bars show the mean value of repeats per species; the FAM strains are marked (red circles).

All completely sequenced *Lactobacillus* genomes (132) were obtained from the NCBI RefSeq database (O'Leary et al., 2016) on December 31, 2016. Genomic repeats were identified as described before (Koren et al., 2013) using Nucmer 3.1 (Kurtz et al., 2004). The number of repeats (longer than 500 bp, sequence identity of 95% or greater) was plotted vs. the maximal repeat length using Seaborn 0.7.1 (https://github.com/mwaskom/seaborn, Figure 1A); two strains were excluded as they were chimeric or could not be processed.

### Bacterial culture and genomic DNA extraction

*L. helveticus* strains FAM8105, FAM22155, and FAM8627 were obtained from the Agroscope culture collection (Agroscope, Liebefeld, Switzerland) and grown under aerobic conditions in 10 mL MRS broth (De Man et al., 1960) at 37°C overnight (see Supplementary Figure 1 for light microscopy images). Bacterial cells were treated with lysozyme (50 mg/mL) for 1 h at 37°C. Genomic DNA (gDNA) was isolated as described elsewhere (Moser et al., 2017), and the concentration determined (Qubit dsDNA BR Assay kit). The gDNA purity was assessed by controlling for RNA contamination using the Qubit RNA HS Assay kit (Thermo Scientific, Massachusetts, USA).

### Genome sequencing and assembly

The gDNA was sequenced on the PacBio RS II platform (three SMRT cells per strain, P6-C4 chemistry, size-selection step (10 kb inserts) with BluePippin; for details, see Supplementary Table 1). Subsequent *de novo* genome assembly using HGAP (Chin et al., 2013) and resequencing steps with Quiver were performed as described before (Remus-Emsermann et al., 2016). Terminal repeats were removed and the genome was circularized using Circlator 1.1.2 (Hunt et al., 2015). Additional rounds of sequence polishing resulted in one complete chromosome and one complete plasmid sequence per strain. FAM8105 and FAM22155 were also sequenced with Illumina MiSeq (paired-end, 2 × 300 bp), and the reads mapped to the polished PacBio assemblies using BWA-MEM (version 0.7.10-r789, Li, 2013). MiSeq reads which did not map to the respective chromosome and plasmid assemblies were assembled with SPAdes (Bankevich et al., 2012) to check for the existence of additional small plasmids.

### Genome coverage exploration of MiSeq data

We compared the genomes assembled only from Illumina MiSeq data vs. the complete PacBio assemblies (**Table 2**). The short read-based genome assemblies were generated with SPAdes (v3.11.0) (Bankevich et al., 2012), requiring a minimum read coverage cutoff of 4 and a minimum contig size of 400 bp (i.e., following recommendations of the SPAdes tutorial). The assembled contigs were mapped to the PacBio assemblies for FAM8105 and FAM22155 with BWA (option -x intractg), files were parsed and the mapping quality was assessed using Samtools (v1.3.1), Bedtools (v2.26.0), and Qualimap (v2.2.1). Subsequently, all coding sequences (CDSs) and pseudogenes in areas with zero coverage were counted and classified as core, accessory or unique gene (see section Comparative Genomics). The circular plot was created with Circos (v0.69-6) (Krzywinski et al., 2009).

### Genome annotation and mining for features of interest

The complete genome sequences were deposited at NCBI GenBank (**Table 2**) and annotated by their Prokaryotic Genome Annotation Pipeline version 3.3 (Tatusova et al., 2016). Putative CRISPR repeats (and their total number) were detected with CRISPRs finder (Grissa et al., 2007) and PILER-CR (Edgar, 2007). Putative Cas proteins were first searched in the NCBI annotation and secondly identified using a Hidden Markov Model (HMM) based search with HMMCAS (Chai et al., 2017). Putative prophages were identified using PHASTER (Arndt et al., 2016), potential genomic islands with Islandviewer 3 (Dhillon et al., 2015); see Supplementary Table 2 for their respective predicted genome positions. All CDSs (without pseudogenes) were compared against the EggNOG 4.5 databases “bactNOG” (bacteria), “bacNOG” (bacilli), “firmNOG” (firmicutes) (Huerta-Cepas et al., 2016), selecting only the hit with the smallest e-value (hits with e-values above 0.001 were not considered) and extracting the respective Clusters of Orthologous Groups (COG) category.

#### GO term enrichment analysis of pseudogenes

First, nucleotide sequences of all annotated pseudogenes of our three strains were extracted, and six-frame translated using transeq (EMBOSS suite 6.6.0.0, http://emboss.open-bio.org/; Rice et al., 2000) to capture potential protein domains present in different reading frames (due to frameshifts). The translated sequences were searched against Pfam database version 31.0 (Finn et al., 2016); hits with e-value smaller than 1e-10 were kept and the corresponding Gene Ontology (GO) terms were extracted if available. An additional Pfam search was performed with the CDS (i.e., intact protein coding genes) from all three strains followed by extraction of GO terms. Using R package “topGO” 2.26.0 (Alexa et al., 2006) from Bioconductor 3.4 (Huber et al., 2015), an enrichment analysis for GO terms of biologic processes (BP) was performed individually per strain (**Table 4**). This was done by using CDSs and pseudogenes combined and their respective GO terms as “gene universe” and pseudogenes as “genes of interest” (Ontology: “BP,” algorithm: “weight01,” statistic: “fisher”; results ranked according to *p*-values). For more details see “topGO” documentation.

#### Selected gene families of interest

We analyzed whether selected gene families involved in amino acid metabolism, or encoding peptide transporters, proteases, and peptidases were encoded in the three FAM, four additional *L. helveticus* strains and in *L. acidophilus* NCFM (Supplementary Tables 8, 9). The analysis was mainly done using blast searches for reference genes or by using the Kyoto Encyclopedia of Genes and Genomes (KEGG) database (for details, see Supplementary Methods in Supplementary Data Sheet 1). The protein sequence of cell envelope protease (CEP, Supplementary Table 8) PrtH3 from strain CNRZ 32 was analyzed with InterPro (https://www.ebi.ac.uk/interpro/ Finn et al., 2017) to retrieve protein domains, which were compared to the predicted *prtH3* pseuodogenes of DPC 4571 and FAM8627. An analysis for peptidoglycan hydrolases (PGH) was done involving all 12 complete *L. helveticus* genomes (**Table 5**) in a similar way. Protein domains of two PGHs were analyzed, the M23 family peptidase of strain H9 and Lysin of strain CNRZ 32. The five copies of 6-Phospho-beta-glucosidases (Supplementary Table 6) and four genes involved in lipid metabolic processes (Supplementary Table 7), both implied by the enrichment analysis of pseudogenes, were also analyzed in more detail for all 12 genomes.

### Phylogenetic analysis

The GenBank records of 24 selected, completely sequenced LAB genomes (see Supplementary Table 3) were downloaded from NCBI RefSeq (March 3rd, 2017). This included 9 *L. helveticus* genomes, 14 other LAB genomes (mostly reference or representative complete genomes; 9 from genus *Lactobacillus* and 5 other LAB) plus *Bacillus subtilis subsp. subtilis* str. 168 as outgroup. The predicted CDSs of all strains, including the three *L. helveticus* strains of this study, were used to calculate a maximum likelihood phylogenetic tree using bcgTree (Ankenbrand and Keller, 2016), which uses HMM models of 107 known housekeeping genes (Dupont et al., 2012). bcgTree was parameterized to perform 100 bootstrap runs while executing RAxML (Stamatakis, 2014), the output (**Figure 4**) was generated using FigTree 1.4.2 (http://tree.bio.ed.ac.uk/software/figtree/).

### Comparative genomics

#### Pan-core genome prediction

GenBank records of the 12 *L. helveticus* genomes (Table 1) were converted to GFF files and analyzed with Roary 3.8.0 (Page et al., 2015) applying standard parameters (minimum blastp identity of 95%) but without paralog splitting. The number of orthologous gene clusters for pan and core genome profiles (**Figure 5A**) and for accessory and unique gene clusters were extracted or calculated from the respective Roary output files or gene presence/absence table. Gene cluster lists for pan, accessory, core and the 12 unique (i.e., strain-specific) genomes are provided as csv files. For every core genome cluster, a multiple sequence alignment of all protein sequences was performed with MUSCLE v3.8.31 (Edgar, 2004) and a profile HMM was calculated using HMMBUILD (HMMER 3.1b2, hmmer.org) with default parameters. We provide one representative sequence per cluster as amino acid (aa) FASTA file and the profile HMM. For pan, accessory and unique genomes we provide just the representative sequences (aa FASTA file). All data files from the comparative genomics analysis are described and summarized in Supplementary Table 10, the data is provided in Supplementary Data Sheet 2.

**Table 1:** Completely sequenced *L. helveticus* strains used in this study and their respective origin.

**Strain**	**Accession**	**Origin**
CAUH18	NZ_CP012381	Isolated from Koumiss (Xinjiang Uighur Autonomous Region, China)
CNRZ 32	NC_021744	Used as industrial cheese starter and cheese flavor adjunct
D76	NZ_CP016827	Ingredient in nutritional supplements “Vitaflor”, isolated from intestine from healthy child (Leningrad, Russia)
DPC 4571	NC_010080	Cheese starter and cheese flavor adjunct isolated from Swiss cheese
H10	NC_017467 (Plasmid: NC_017468)	Isolated from traditional fermented milk (Shigatse City of Tibet, China)
H9	NZ_CP002427	Isolated from kurut (Nagqu County of Tibet, China)
KLDS1.8701	NZ_CP009907 (Plasmid: NZ_CP009908)	Isolated from sour milk (Sinkiang, China)
MB2-1	NZ_CP011386	Isolated from fermented milk (Baicheng, southern Xinjiang, China)
R0052	NC_018528 (Plasmid: NC_014386)	Probiotic strain isolated from sweet acidophilus milk (France)
**FAM8105**	CP015496 (Plasmid: CP015497)	Isolated from raw milk (Thurgau, Switzerland)
**FAM22155**	CP015498 (Plasmid: CP015499)	Isolated from natural whey culture (Luzern, Switzerland)
**FAM8627**	CP015444 (Plasmid: CP015445)	Isolated from dairy product (not further specified) (Switzerland)

*The three strains sequenced in this study are listed at the bottom (in bold). Data source: NCBI RefSeq (as of Feb 28, 2017). Strain MTCC 5643 (Prajapati et al., 2011) is not completely sequenced (its 50 contigs can be downloaded from the NCBI); it was thus not included in our comparison*.

#### COG categories

For clusters of core and accessory genome, as well as the unique genes, a representative sequence was extracted and the COG category was determined as described above.

### Metagenome sequencing and analysis

DNA from a NWC was extracted as described elsewhere (Moser et al., 2017), DNA libraries were prepared (TruSeq DNA PCR-Free LT Library Prep Kit; insert size: 350 bp) and sequenced on an Illumina HiSeq 3000 (paired-end, 2 × 151 bp). Reads were assembled using SPAdes 3.9.0 (Bankevich et al., 2012). Performing a blastn search of the resulting contigs against NCBI RefSeq, we first determined the genomes (and plasmids, where available) with most hits, requiring that at least 95% of the raw metagenome reads were assigned. This implied 8 *L. helveticus*, 7 *S. thermophiles*, and one *L. delbrueckii* RefSeq strain. The reads were then mapped to these genome sequences plus our three FAM strains using BWA-MEM (version 0.7.15-r1140 Li, 2013; using option -a); the resulting SAM file was filtered to remove non mapping reads and supplementary alignments. To determine a qualitative species level distribution, we counted how many reads mapped to genomes of respective species; reads mapping to several targets were attributed in a proportional fashion. As few reads mapped to more than one species, we did not correct for different numbers of target genomes. As all genomes had roughly the same size we did not correct for genome size either. Next, we counted reads that mapped exclusively either to the three FAM or to NCBI *L. helveticus* genomes. To do this, the resulting SAM file from the previous steps was first parsed and a correction for the different number of target genomes (three FAM, eight NCBI strains) was performed. In a last analysis step, we determined how many of the uniquely mapping reads (i.e. reads just mapping to one target sequence) mapped to the FAM and NCBI *L. helveticus* genomes, respectively.

## Results

### Determining the genome assembly difficulty of *Lactobacillus* and *L. helveticus*

Aiming to increase the number of completely sequenced *L. helveticus* strains, we first explored the repeat structure of all *Lactobacillus* strains for which complete genome sequences have been deposited in NCBI's RefSeq. The total number of repeats and the length of the longest repeat of bacterial genomes are two parameters that have a profound impact on their genome assembly complexity (Koren et al., 2013).

Using an in-house software prototype, we calculated the overall number of repeats above 500 bp and >95% identity identified by Nucmer (Kurtz et al., 2004) vs. the length of the overall longest repeat (see Methods) for 130 completely sequenced *Lactobacillus* strains. This analysis resulted in the classification of LAB genomes into three classes with increasing demands for assembly due to the number and size of the repeats. Most genomes are classified as class I genomes (*N* = 57, 43.8%) that are straight-forward to assemble with PacBio long reads as they harbor few repeats, with the longest repeat representing multi-copy rDNA operons typically around 6–7 kb in length (Koren et al., 2013; Figure 1A). Furthermore, 32 (24.6%) class II genomes were observed that are characterized by the presence of a large number (more than 100) of repeats (500 bp to several kb), but none larger than the rDNA operon. Using data from the PacBio RSII platform, such genomes should also be straightforward to be *de novo* assembled into complete genome sequences; in contrast, relying on only Illumina short reads would produce tens to hundreds of contigs. Finally, we also noted a sizable fraction of class III genomes (41, 31.5%; Figure 1A). Due to their long, almost identical repeats well above 6–7 kb, these genomes can be extremely difficult to assemble. Such repeats can be resolved only with very long reads, which can be obtained by including a size selection step in the library preparation.

We next specifically assessed *L. helveticus* strains, for which nine complete genomes were available at NCBI's RefSeq (blue circles, Figure 1A). Eight of these strains were isolates from the dairy environment, while strain D76 was described as acting as an ingredient in nutritional supplements (Table 1). Eight of the genomes are class II genomes (Figure 1A), while strain H10 (Zhao et al., 2011) is classified as a class III genome and harbors long, nearly identical repeats >30 kb.

Finally, an analysis of the predominant assembly complexity classification for different *Lactobacillus* species indicated that *L. helveticus* strains had the second highest mean repeat number among all *Lactobacillus* species, outnumbered only by *L. backii* (Geissler et al., 2016) (Figure 1B). More importantly, due to the overall higher percentage of class III genomes, *L. delbrueckii* (73%), *L. backii* (60%), *L. casei* (50%), *L. plantarum* (43%), and *L. fermentum* (40%) represent *Lactobacillus* species that may pose considerable challenges for researchers aiming to carry out complete genome assembly projects similar to that we describe here for *L. helveticus*.

### Genome sequencing, assembly and annotation of three *L. helveticus* isolates

We selected three *L. helveticus* strains (FAM8105, FAM22155, and FAM8627) from the Agroscope culture collection, which originated from different dairy products (including raw milk and natural whey cultures, Table 1), and sequenced them on the PacBio RSII platform. To obtain a high sequence coverage, we used three single-molecule, real-time (SMRT) cells per strain, and to possibly even completely assemble class III genomes we used the BluePippin size selection protocol (see Methods). A high coverage would allow us to rely on algorithms that remove the random errors of PacBio reads (Chin et al., 2013) and to obtain completely sequenced genomes of high quality.

This *de novo* genome assembly approach resulted in one completely assembled chromosome and one complete plasmid for each strain, with a PacBio sequence coverage above 150-fold except for the plasmid of strain FAM22155 (Table 2). This is most likely because this plasmid is relatively small (7.5 kbp) and thus selected against in the BluePippin size selection step (see Methods). MiSeq short read data (available for FAM8105 and FAM22155) were used to check the quality of the assembly and to eliminate potential single nucleotide mis-assemblies reported for PacBio data (Laehnemann et al., 2015). Although no evidence for any mis-assembly was found for strain FAM22155, FAM8105 harbored a single nucleotide deletion in a homopolymer stretch in the chromosome and the plasmid (data not shown), which were corrected. This confirms that our assemblies are of a high quality. In addition, we used the MiSeq data to search for small plasmids potentially missed due to the size selection step during library preparation. However, no evidence for additional small plasmids could be found. Finally, a repeat analysis of the complete genomes of FAM8105, FAM22155, and FAM8627 classified all three as class II genomes (red circles, Figure 1A).

**Table 2:** Genome statistics of our three completely sequenced *L. helveticus* strains.

	**FAM8105**	**FAM22155**	**FAM8627**
**No. of chromosomes**	**1**	**1**	**1**
Length	2,209,387 bp	2,191,149 bp	2,035,631 bp
GC content	37.1%	37.1%	37.0%
Total genes	2,217	2,178	2,057
CDSs/RNAs	1,876/76	1,849/78	1,691/78
Pseudogenes	265	251	288
Transposases	194	192	130
Repeat info: max. length/No. repeat pairs (class)	5,367/341 (class II)	5,466/247 (class II)	5,466/198 (class II)
Average coverage PacBio	511 x	354 x	227 x
Average coverage MiSeq	152 x	456 x	(no MiSeq data)
No. of detected IS elements^*^ (TnpPred)	157	161	112
No. of CRISPR clusters	1	0	1
Cas proteins	Yes	No	Yes
Phages (Supplementary Table 2)	2 intact, 1 questionable, 1 incomplete	1 intact	1 incomplete
GenBank accession	CP015496	CP015498	CP015444
**No. of plasmids**	**1**	**1**	**1**
Length	45,858 bp	7,514 bp	13,399 bp
GC content	34.2%	35.1%	34.8%
Total genes (all CDSs)	43	10	14
Average coverage PacBio	659 x	38 x	155 x
Average coverage MiSeq	471 x	3316 x	(no MiSeq data)
Phages	No	No	No
GenBank accession	CP015497	CP015499	CP015445

* *See Supplementary Results in Supplementary Data Sheet 1, Supplementary Table 4, Supplementary Figure 4*.

**Figure 2:**
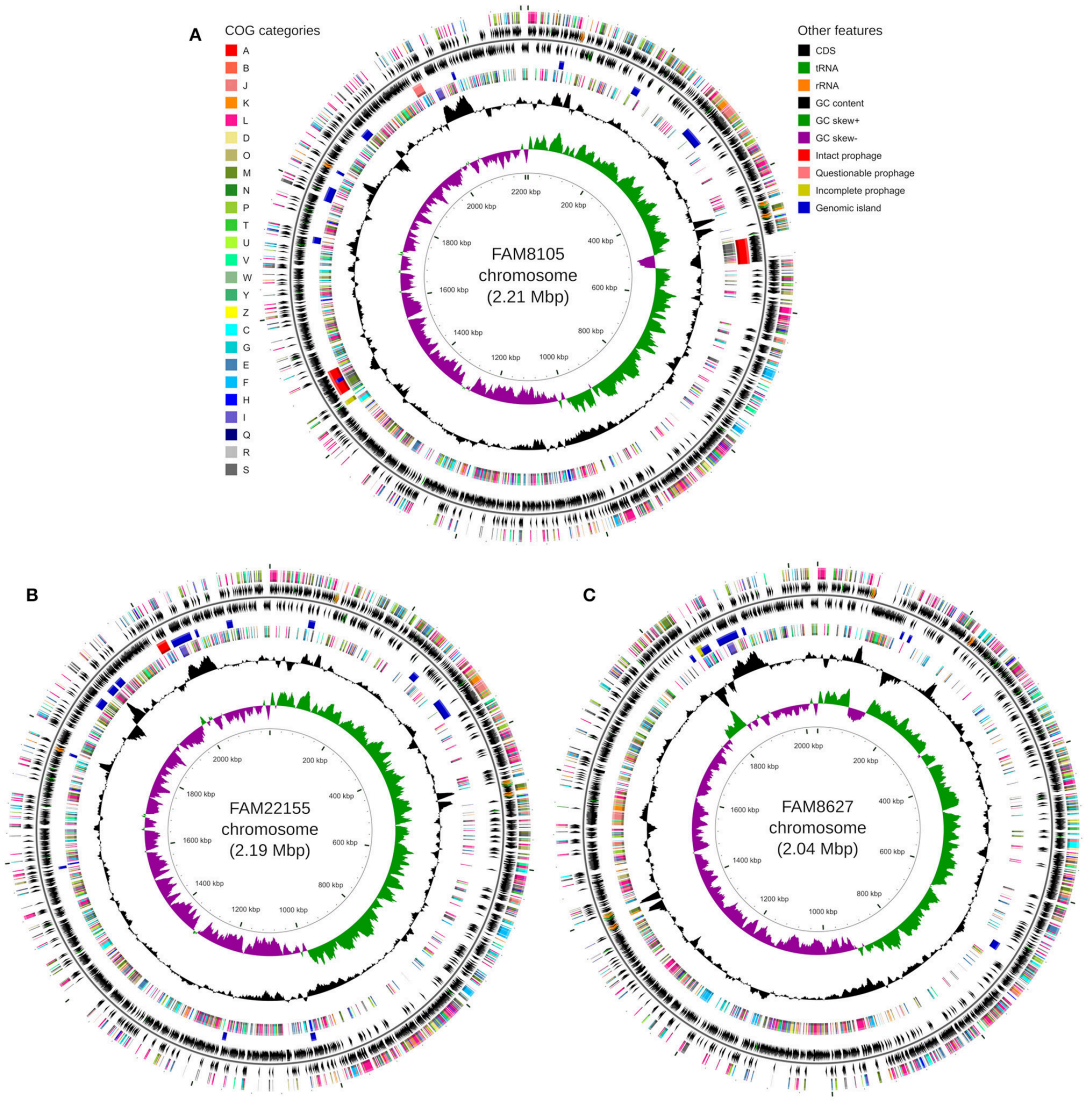
Circular genome map for FAM8105 **(A)**, FAM22155 **(B)**, and FAM8627 **(C)**, generated using CGView (Stothard and Wishart, 2005). For every sub-figure, the following features are shown (moving from the outermost track inwards, origin of replication is positioned at 0 kbp): (1) CDS on forward strand colored according to COG category, (2) CDS (*black*), tRNA (*green*) and rRNA (*orange*) on forward strand, (3) black line representing genome sequence, (4) CDS (*black*), tRNA (*green*) and rRNA (*orange*) on reverse strand, (5) Intact prophages (*red*), questionable prophages (*light red*), incomplete prophages (yellow) and genomic islands (*blue*), (6) CDS on reverse strand colored according to COG category, (7) GC content (*black*), (8) positive and negative GC skew (*green* and *purple*, respectively), and (9) genome position in kbp.

The genomes were annotated at NCBI (see Methods). A detailed summary of their genome statistics is shown in Table 2. An overview of their genome features, including CDSs, rRNAs, and tRNAs, is shown for chromosomes (Figure 2) and plasmids (Supplementary Figure 2). The distribution of functional COG categories (see Methods; Table 3) was similar for all three *L. helveticus* strains. However, compared to annotation projects we carried out in the past (data not shown), we observed a high number of CDS classified in category “L” (“Replication, recombination and repair”). This has been reported previously for the genus *Lactobacillus* (Lukjancenko et al., 2012) and can, at least in part, be attributed to the many transposase sequences in the *L. helveticus* genomes which get classified as class “L.” For FAM8105, 56.9% of the class “L” hits were transposases (194/341), for FAM22155, 56.3% (192/341) and for FAM8627, 47.6% (130/273) (Table 2). Accordingly, among the COG categories for which a function could be assigned, category L contained the largest number of genes (Table 3).

**Table 3:** Number of genes associated with COG functional categories for all three sequenced strains, and for core, accessory and unique genome.

**Class**	**FAM8105**	**FAM22155**	**FAM8627**	**Core genome**	**Accessory genome**	**Unique genome**
J, Translation, ribosomal structure and biogenesis	134 (7.0%)	132 (7.1%)	133 (7.8%)	125 (12.7%)	10 (0.8%)	1 (0.1%)
K, Transcription	116 (6.0%)	104 (5.6%)	104 (6.1%)	61 (6.2%)	90 (7.0%)	63 (5.9%)
L, Replication, recombination and repair	341 (17.8%)	341 (18.3%)	273 (16.0%)	83 (8.4%)	164 (12.8%)	79 (7.4%)
[Annotated as transposase]	[194 (10.1%)]	[192 (10.3%)]	[130 (7.6%)]			
D, Cell cycle control, Cell division, chromos. partitioning	22 (1.1%)	26 (1.4%)	22 (1.3%)	17 (1.7%)	8 (0.6%)	12 (1.1%)
V, Defense mechanisms	42 (2.2%)	47 (2.5%)	29 (1.7%)	11 (1.1%)	58 (4.5%)	51 (4.8%)
T, Signal transduction mechanisms	29 (1.5%)	30 (1.6%)	28 (1.6%)	21 (2.1%)	13 (1.0%)	11 (1.0%)
M, Cell wall /membrane biogenesis	84 (4.4%)	86 (4.6%)	84 (4.9%)	56 (5.7%)	48 (3.8%)	89 (8.3%)
N, Cell motility	3 (0.2%)	3 (0.2%)	3 (0.2%)	3 (0.3%)	0 (0.0%)	0 (0.0%)
U, Intracellular trafficking and secretion	17 (0.9%)	18 (1.0%)	16 (0.9%)	15 (1.5%)	2 (0.2%)	0 (0.0%)
O, Posttranslational modification, protein turnover, chaperones	46 (2.4%)	48 (2.6%)	44 (2.6%)	34 (3.4%)	23 (1.8%)	5 (0.5%)
C, Energy production and conversion	47 (2.4%)	54 (2.9%)	47 (2.8%)	38 (3.8)	41 (3.2%)	14 (1.3%)
G, Carbohydrate transport and metabolism	103 (5.4%)	97 (5.2%)	85 (5.0%)	63 (6.4%)	78 (6.1%)	25 (2.3%)
E, Amino acid transport and metabolism	91 (4.7%)	102 (5.5%)	96 (5.6%)	54 (5.5%)	80 (6.3%)	27 (2.5%)
F, Nucleotide transport and metabolism	81 (4.2%)	80 (4.3%)	80 (4.7%)	47 (4.8%)	61 (4.8%)	5 (0.5%)
H, Coenzyme transport and metabolism	35 (1.8%)	34 (1.8%)	32 (1.9%)	23 (2.3%)	17 (1.3%)	8 (0.7%)
I, Lipid transport and metabolism	38 (2.0%)	36 (1.9%)	34 (2.0%)	30 (3.0%)	10 (0.8%)	2 (0.2%)
P, Inorganic ion transport and metabolism	64 (3.3%)	70 (3.8%)	65 (3.8%)	47 (4.8%)	35 (2.7%)	6 (0.6%)
Q, Secondary metabolites biosynthesis, transport and catabolism	6 (0.3%)	4 (0.2%)	4 (0.2%)	2 (0.2%)	5 (0.4%)	1 (0.1%)
S, Function unknown	487 (25.4%)	444 (23.9%)	408 (23.9%)	229 (23.2%)	383 (30.0%)	383 (35.8%)
Not in COG category	133 (6.9%)	103 (5.5%)	118 (6.9%)	29 (2.9%)	152 (11.9%)	287 (26.8%)
Total CDS	1,919 (100%)	1,859 (100%)	1,705 (100%)	988 (100%)	1,278 (100%)	1,069 (100%)

*COG categories which contained no genes/gene clusters are not shown. Numbers in brackets show percentage as fraction of total number of CDSs*.

### Comparison of short and long read-based assemblies with respect to gene coverage

**Figure 3:**
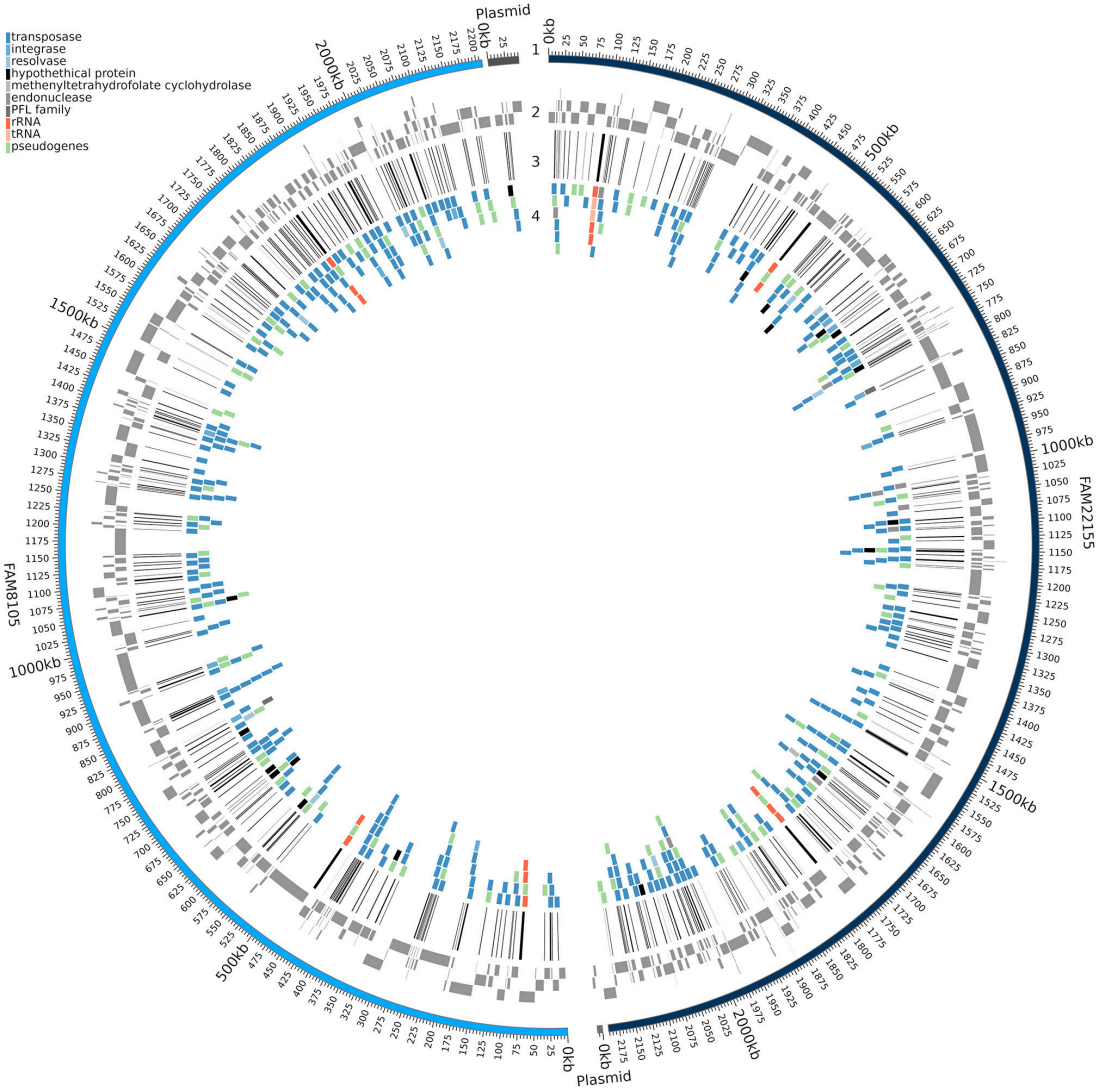
Genomic regions missed in the assembly based on MiSeq reads. A circular plot of FAM8105 and FAM22155 is shown, where the outer ring (1) represents the complete PacBio assembly of FAM8105 (light blue) and FAM22155 (dark blue) along with their plasmids. Moving inwards, the contigs of the MiSeq assembly are shown next (gray boxes, 2), then lines that illustrate the coverage gaps in the MiSeq assemblies (black, 3), and finally genes which are affected by the gaps in the MiSeq assembly (4; their colors correspond to the legend in the top left).

Despite the value of complete genome sequences, they are still highly underrepresented in public sequence repositories. This is also true for LAB: Among the 213 strains recently analyzed, almost all the genome assemblies (> 96%) were fragmented (Sun et al., 2015). The number of contigs reported for the LAB strains ranged from 8 to 964 (a median of about 90). A comparison of the FAM8105 and FAM22155 genomes assembled only from Illumina MiSeq data using SPAdes vs. the respective final PacBio-based reference genomes provided an insight into the extent of such differences (Figure 3): About 230 regions with an average size of roughly 1,000 bp were not covered by the MiSeq assemblies, which amounted to 10–12% of the actual genome of these two strains (genome size around 2.2 Mbp; Table 2). Notably, for the repeat-rich *L. helveticus* genomes, around 10% of the annotated CDSs would be missed in addition to the rRNA operons that represent the largest repeats in the genome, i.e., roughly 200 genes (Table 2). For pseudogenes, the percentage of missed cases was even higher, surpassing 20% of all pseudogenes.

### Phylogenetic tree with a focus on *L. helveticus*

**Figure 4:**
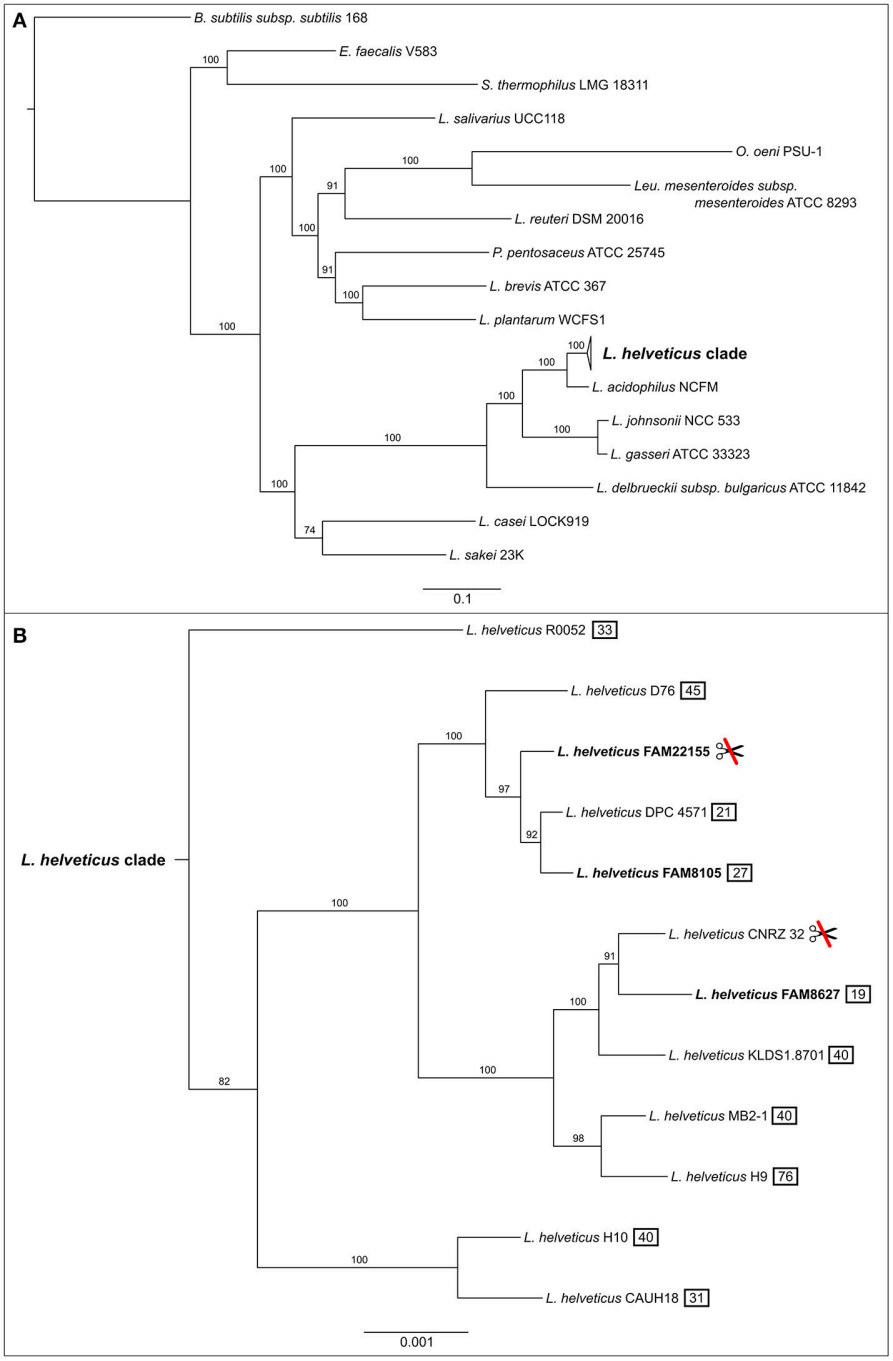
Maximum likelihood phylogenetic tree of completely sequenced *L. helveticus* strains, in the context of several key LAB strains. Phylogenetic tree was constructed using a concatenated alignment of 107 known housekeeping genes (Dupont et al., 2012). **(A)** The collapsed *L. helveticus* clade relative to other LAB bacteria. Bootstrap scores for all nodes are shown (percentage of 100 bootstrap runs). The bar at the bottom represents the number of amino acid substitutions per site. *Bacillus subtilis* subsp*. subtilis* 168 served as outgroup. **(B)** Expanded *L. helveticus* clade based on the same calculation as above. The three strains of this study are shown in bold. The *L. helveticus* clade has a 100 times higher resolution than the complete tree which is reflected by the bar. The symbol showing crossed scissors indicates two strains without a CRISPR/Cas system; the numbers in a black box indicate how many CRISPR spacers were detected in total per strain.

To ensure reliable phylogenetic placement of the newly sequenced strains, we selected several taxonomically diverse LAB genomes on top of all completely sequenced *L. helveticus* genomes from NCBI (Supplementary Table 3). The maximum likelihood phylogenetic tree generated from these genomes is based on 107 known housekeeping genes and has very good bootstrap support (Figure 4). The three newly sequenced strains form a monophyletic clade with the other nine *L. helveticus* strains (Figure 4A), which is clearly separated from the other groups and is characterized by a relatively low degree of intra-clade variation. As reported previously, the genus *Lactobacillus* is not monophyletic, but paraphyletic (Mayr and Bock, 2002; Sun et al., 2015); i.e., the *Lactobacillus* clade also includes *Leuconostoc, Oenococcus*, and *Pediococcus* species.

The three FAM strains fall into two different subgroups within the *L. helveticus* clade (Figure 4B). FAM8627 is close to CNRZ 32 (Christiansen et al., 2008; Broadbent et al., 2013), a strain used as a starter culture and for the production of bioactive peptides in milk. FAM8105 and FAM22155 are more closely related to DPC 4571 (Hannon et al., 2003; Callanan et al., 2008), another strain known to be beneficial in cheese production. Of note, strains FAM22155 and CNRZ 32 seem to lack a CRISPR/Cas system as neither CRISPR repeats nor Cas proteins were detected (Figure 4B).

### Comparative genomics of 12 *L. helveticus* strains

To the best of our knowledge and as noted in a recent review (Stefanovic et al., 2017), no pan-core genome study has been reported for *L. helveticus*. Thus, we carried out such an analysis on the 12 complete genomes using Roary (Page et al., 2015). The pan genome is generally defined as the sum of all genes in a species, whereas the core genome is defined as the orthologous genes that are present in all strains of a species (Medini et al., 2005). Furthermore, the accessory genome comprises orthologous gene clusters (orthologous genes from here on are referred to as “gene clusters” or “genes,” depending on the context) that, in our example, are found in at least two and up to 11 strains. Finally, we also determined genes that occur in only one of the strains; these genes represent the unique or “strain-specific” genome.

**Figure 5:**
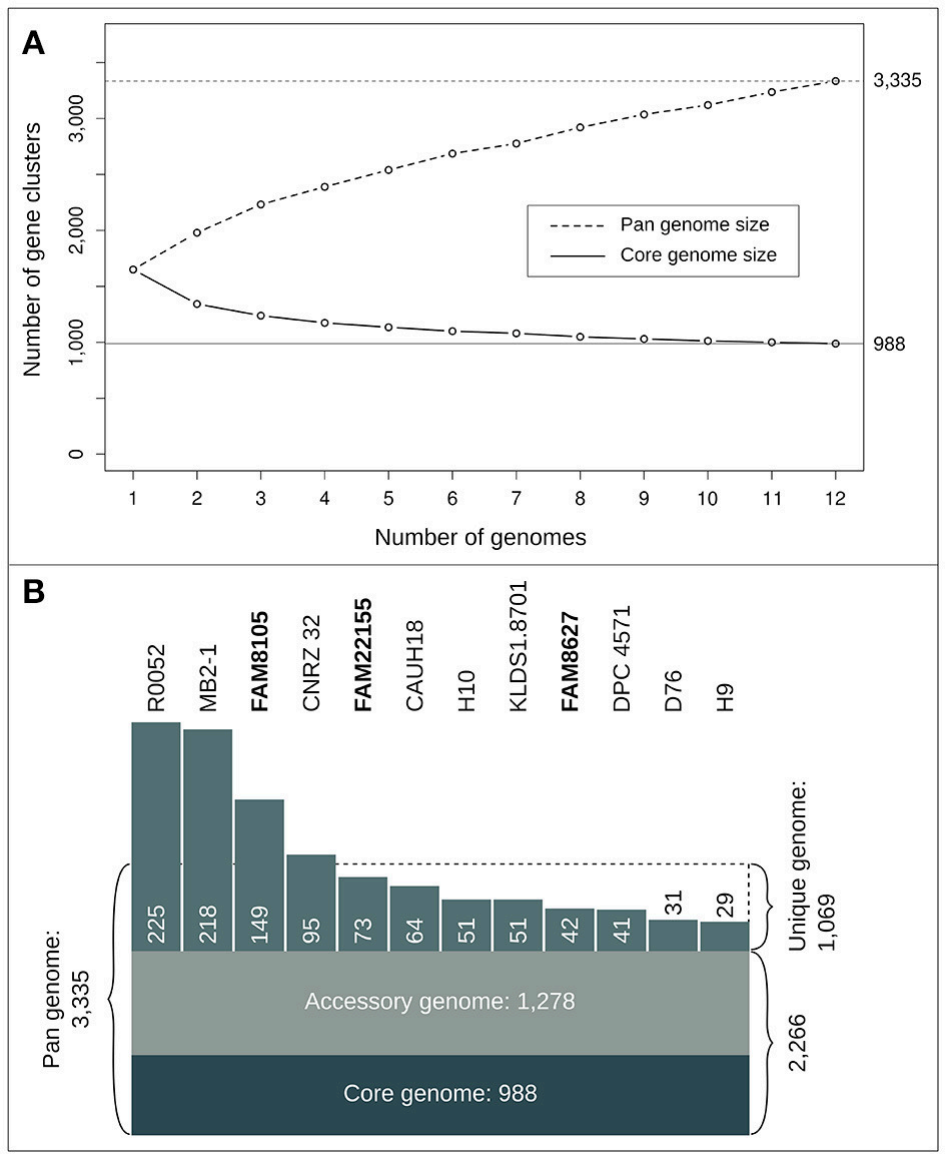
Statistics of the *L. helveticus* pan- and core genome based on 12 completely sequenced strains. **(A)** Number of gene clusters for pan (dashed line) and core genome profile (straight line) when successively adding genomes. The curves show mean values of 10 iterations where genomes were randomly added and the pan and core genome was calculated successively for all 12 steps. **(B)** Number of gene clusters (reflected by the size of the respective areas) for core, accessory, pan and unique genome.

As more genomes were added, the size of the core genome diminished and reached 988 gene clusters for all 12 genomes (Figure 5A). The curve of the pan genome hints at a still “open” pan genome, comparable to results from a similar-sized pan-core genome study for 17 *L. casei* genomes (Broadbent et al., 2012). Overall, we identified a pan genome of 3,335 gene clusters (Figure 5B). They could be further divided into a core genome of 988 gene clusters (29.6%) present in all 12 strains, an accessory genome of 1,278 gene clusters (38.3%) present in a subset of the 12 strains (Supplementary Figure 3), and a total of 1,069 strain-specific genes (32.1%). The number of strain-specific gene clusters ranged from 29 genes only found in strain H9 up to 225 gene clusters unique to strain R0052 (Figure 5B). Notably, among the CDSs missed in the two MiSeq assemblies (Figure 3), not only accessory genes were missed, but also around 30 core genes, i.e., roughly 3% of the close to 1,000 core genes identified.

To identify whether gene clusters of certain functional groups are preferentially found in all 12 strains (core genome) or if they diverge more between the strains, COG functional categories for core, accessory and unique genome of all 12 *L. helveticus* strains were explored (Table 3; see Methods). As expected, the COG functional categories of some highly conserved biological processes, such as “Translation, ribosomal structure, and biogenesis” (Class J), and “Intracellular trafficking and secretion” (Class U), were enriched among the core genes and depleted in the unique genome. In contrast, the category K “Transcription” was equally present in the core, accessory and unique genomes, indicative of specific transcriptional regulatory mechanisms in some of the strains. For the class “Replication, recombination, and repair” (L), enrichment in the accessory genome was observed. As mentioned, this is mainly caused by transposases which are often found in more than one of the 12 strains but not in all. Enrichment among accessory and unique genes was observed for “Defense mechanisms” (Class V). Importantly, for the unique genes, we observed enrichment of genes in the COG functional category “Cell wall/membrane biogenesis” (Class M). Finally, genes of unknown function (Class S) were enriched in the unique genome, as well as genes not assigned to any COG class. For the latter, the largest difference between their presence in the core genome (very low) vs. the unique genome could be detected (Table 3).

### Genome mining for pseudogenes and gene families of interest

#### GO term enrichment analysis of pseudogenes

Enrichment of certain genomic features can help to elucidate lifestyle adaptations, such as the adaptation to a new biological niche (D'Souza and Kost, 2016). To analyze whether certain biological processes were overrepresented among the large number of annotated pseudogenes (Table 2), we tested for enrichment of GO terms in the pseudogenes vs. a background distribution of GO terms in the CDSs plus pseudogenes of the three FAM *L. helveticus* genomes (see Methods).

GO terms associated with amino acid membrane transport, carbohydrate and lipid metabolic processes were overrepresented among the pseudogenes of the three FAM strains (Table 4), which can be explained with an adaptation to the nutrient-rich milk environment. The inactivation of genes involved in amino acid biosynthesis in such an environment is well-known (Makarova et al., 2006; Callanan et al., 2008; Christiansen et al., 2008; Cremonesi et al., 2013). The genes for 6-phospho-beta-glucosidases are grouped under the GO term “carbohydrate metabolic process” (GO:0005975); their encoded enzymes are associated with the hydrolysis of glucosidic bonds and contain the InterPro domain “glycoside hydrolase family 1.” All five share high similarity (Supplementary Table 6): Three gene products are just below 500 amino acids in length, one member is annotated as a pseudogene that covers the N-terminal 211 aa, while the last member encodes a short 48 aa protein covering the C-terminal part of the three longer proteins. Exploring these five members in all 12 complete genomes, we found that three were inactivated not only in the three FAM strains, but also in several other completely sequenced *L. helveticus* strains (Supplementary Table 6).

**Table 4:** GO terms overrepresented among the pseudogenes in our three *L. helveticus* strains.

**FAM8105**	**FAM22155**	**FAM8627**
Amino acid transmembrane transport; *p*-value = **0.012** (GO:0003333)	Carbohydrate metabolic process; *p*-value = **0.0031** (GO:0005975)	Amino acid transmembrane transport; *p*-value = **0.0025** (GO:0003333)
Lipid metabolic process; *p*-value = **0.026** (GO:0006629)	Amino acid transmembrane transport; *p*-value = **0.0483** (GO:0003333)	Transposition, DNA-mediated; *p*-value = **0.0081** (GO:0006313)
Transmembrane transport; *p*-value = **0.026** (GO:0055085)	Lipid metabolic process; *p*-value = 0.0735 (GO:0006629)	Lipid metabolic process; *p*-value = **0.0431** (GO:0006629)
Carbohydrate metabolic process; *p*-value = 0.098 (GO:0005975)	Regulation of transcription; DNA-templated; *p*-value = 0.0970 (GO:0006355)	DNA recombination; *p*-value = 0.0579 (GO:0006310)
Glutamine metabolic process; *p*-value = 0.099 (GO:0006541)	Glutamine metabolic process; *p*-value = 0.097 (GO:0006541)	Phosphoenolpyruvate-dependent sugar phosphotransferase; *p*-value = 0.0711 (GO:0009401)
Terpenoid biosynthetic process; *p*-value = 0.099 (GO:0016114)	Terpenoid biosynthetic process; *p*-value = 0.097 (GO:0016114)	Isoprenoid biosynthetic process *p*-value = 0.0795 (GO:0008299)

*The six terms with lowest p-values (see Methods) are shown for each strain. Values below a significance level of 0.05 are shown in bold*.

Finally, enrichment for lipid metabolic processes could hint at changes in enzymes that may affect the lipid composition of the cell membrane. An overview of four selected gene products belonging to this class is shown in Supplementary Table 7, three of which were inactivated in a subset of the strains.

#### Genes relevant for cheese ripening

Protein degradation, amino acid catabolism, and autolysis are major biochemical processes taking place during cheese ripening. Thus, we took a closer look at genes associated with these processes and analyzed the three newly sequenced FAM strains, four strains that broadly cover the *L. helveticus* clade (including DPC 4571, CNRZ 32, H10, and R0052; Figure 4B) and the *L. acidophilus* strain NCFM (Supplementary Tables 8, 9).

**Figure 6:**
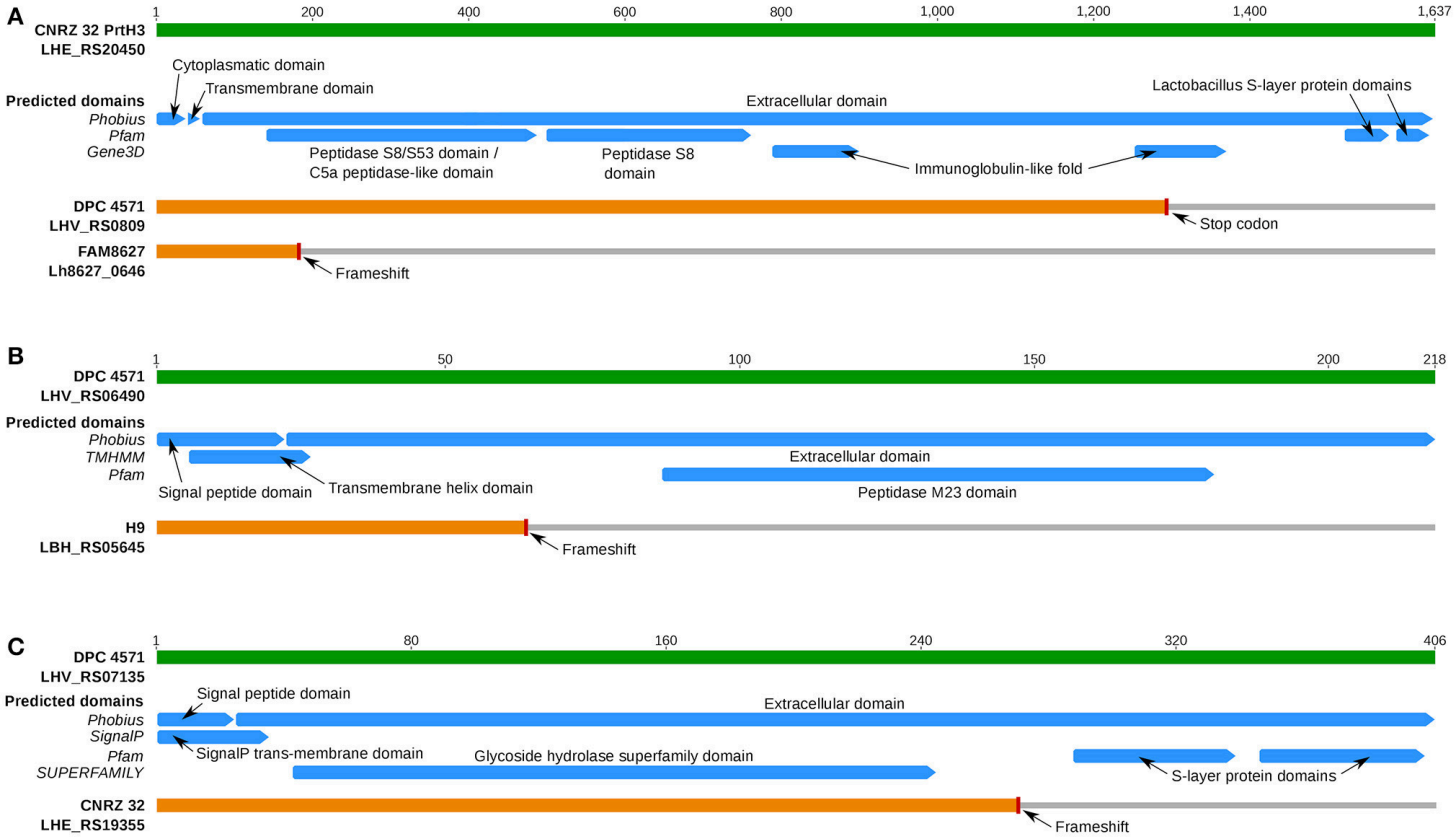
Overview of predicted InterPro protein domains of selected cell membrane proteins of interest. **(A)** Protein domains for CEP PrtH3 (blue bars; prediction source on the left). The green bar represents PrtH3 of strain CNRZ 32. The two predicted *prtH3* pseudogenes from DPC 4571 (premature stop codon predicted close to C-Terminus, 99.3% aa identity until stop codon to DPC 4571) and FAM8627 (100% aa identity until frameshift close to N-terminus) are shown in orange. **(B)** Protein domains for a PGH gene inactivated only in *L. helveticus* strain H9. The green bar represents the intact PGH with a M23 family peptidase domain (Locus tag LHV_RS06490) of strain DPC 4571. The frameshift in the N-terminus (aa position 64) of the ortholog in strain H9 is predicted to inactivate protein function as the protein no longer contains the peptidase M23 domain. **(C)** Protein domains for a PGH gene inactivated only in strain CNRZ 32. The green bar represents the intact PGH with a glycoside hydrolase domain (Locus tag LHV_RS07135) of strain DPC 4571. The frameshift occurs at aa position 274 and thus likely inactivates the original function of the protein since the two SLAP domains are in the frame-shifted region.

All three FAM strains and the dairy isolates DPC 4571 and CNRZ 32 harbored the gene for the cell membrane-localized CEP PrtH3 (Supplementary Table 8). However, a frameshift that likely renders the protein inactive was present in the N-terminal coding region of *prtH3* in FAM8627 (Figure 6A). This strain encodes two additional CEPs named PrtH1 and PrtH4. These CEPs are also present in CNRZ 32, which possesses a total of four CEP-encoding genes, but they are absent in the other strains (Supplementary Table 8). To address which of the CEPs could possess an important function in *L. helveticus*, we measured proteolytic activity in the cell wall fraction (see Supplementary Methods in Supplementary Data Sheet 1) of the three FAM strains. FAM8627 did not exhibit any detectable proteolytic activity, indicating that PrtH3 was the predominant CEP present in the cell wall extracts used here (Supplementary Table 5; Supplementary Methods in Supplementary Data Sheet 1) and providing a genotype-phenotype link (Figure 6A). The *prtH3* gene of DPC 4571 carried a stop codon in the C-terminal coding region, and thus, likely encodes a functional protein lacking the S-layer domains and an immunoglobulin domain, potentially affecting additional functions of the protein or interaction(s) with other proteins (Figure 6A).

The GO term enrichment analysis of the pseudogenes had revealed that amino acid membrane transport was one process affected in the FAM strains (Table 4), i.e., a function not needed in a nutrient-rich milk environment. This observation is further supported by the fact that two or more copies of several peptide transporter operons are encoded in the respective genomes (Supplementary Table 8). All analyzed strains (except FAM8105 and H10) carried genes encoding several oligopeptide transporter operons and the peptidase complement (Supplementary Table 8). Amino acid proto- and auxotrophies have been reported for strain CNRZ 32 (Christiansen et al., 2008). Therefore, we compared the gene products associated with amino acid metabolism of this strain with the eight strains mentioned above (Supplementary Table 9A). The analyzed strains did not possess complete biosynthetic pathways for Arg, Glu, His, Ile, Leu, Lys, Phe, Pro, Thr (except strain H10), Trp, Tyr, and Val.

Researchers have suggested that variations in the autolytic potential of *L. helveticus* strains are linked to either differences in the set of peptidoglycan hydrolases (PGHs) (Jebava et al., 2011) and/or the cell wall composition (Vinogradov et al., 2013). Based on PCR experiments, Jebava and colleagues proposed that nine peptidoglycan hydrolases are ubiquitous genes in *L. helveticus*. In contrast to these results, we identified only five of these nine genes in the functional core genome (Table 5). These five gene products were intact in all strains and were highly conserved to their DPC 4571 ortholog (>98% average pairwise aa identity, Table 5). In contrast, the remaining genes were inactivated in one or several strains; therefore, those genes were designated as pseudogenes in the annotation and thus, are not part of the core genome. Due to frameshifts, two genes were annotated as pseudogenes in one of the 12 strains. The ortholog of LHV_RS06490 in strain H9 contained a frameshift in the N-terminal third of the encoded protein, which is predicted to abrogate its function (Figure 6B). For the ortholog of LHV_RS07135 in strain CNRZ 32, the frameshift in the C-terminal third would leave out two S-layer protein domains, and thus, may have a more subtle effect (Figure 6C). However, the frameshift could also affect important interactions with other proteins or carbohydrates and affect attachment to the cell envelope (Hynönen and Palva, 2013). The two remaining genes (LHV_RS06550 and LHV_RS10160) showed more variability among the strains: Mutations that likely lead to inactive gene products were observed in four (LHV_RS06550) and nine strains (LHV_RS10160), respectively (Table 5). In summary, the data indicate that there is considerable variability concerning the relevant genes, including proteins at the cell surface.

**Table 5:** Overview of peptidoglycan hydrolase (PGH) genes in complete *L. helveticus* genomes.

**Strain**	**LHV_RS00930 N-acetylmuramidase**	**LHV_RS00935 Amidase**	**LHV_RS02820 N-acetylmuramidase**	**LHV_RS03290 Lysozyme**	**LHV_RS05260 N-acetylmuramidase**	**LHV_RS06490 M23 family peptidase**	**LHV_RS07135 Lysin**	**LHV_RS06550 M23 family peptidase**	LHV_RS10160 ^*1^ Lysin
FAM8105	✓	✓	✓	✓	✓	✓	✓	✓	P (FS)
FAM22155	✓	✓	✓	✓	✓	✓	✓	✓	P (FS)
FAM8627	✓	✓	✓	✓	✓	✓	✓	✓	P (FS)
CAUH18	✓	✓	✓	✓	✓	✓	✓	P (ST)	✓
CNRZ 32	✓	✓	✓	✓	✓	✓	P (FS)	✓	P (FS)
D76	✓	✓	✓	✓	✓	✓	✓	✓	P (FS)
DPC 4571	✓	✓	✓	✓	✓	✓	✓	✓	P (FS)
H10	✓	✓	✓	✓	✓	✓	✓	P (ST)	✓
H9	✓	✓	✓	✓	✓	P (FS)	✓	P (FS)	P (FS)
KLDS1.8701	✓	✓	✓	✓	✓	✓	✓	✓	P (FS)
MB2-1	✓	✓	✓	✓	✓	✓	✓	P (FS)	P (FS)
R0052	✓	✓	✓	✓	✓	✓	✓	✓	✓
In core genome	Yes	Yes	Yes	Yes	Yes	No	No	No	No

*The locus tags of nine PGH genes of strain DPC 4571 are shown on top along with the annotation of the encoded protein. The nine genes were selected based on a detailed study of PGHs (Jebava et al., 2011). PGHs annotated as functional are marked with a tick mark (✓), pseudogenes are marked with “P”, the reason for the pseudogene annotation is shown in brackets. Five of the nine PGH genes belonged to the core genome and were highly conserved (>98% average pairwise aa identity, using BLSM62 substitution-scoring matrix)*.

*P (ST): Pseudogene with premature stop codon*.

*P (FS): Pseudogene with frameshift*.

*1 *Frameshift according to RefSeq annotation*.

### Metagenome analysis of cheese starter cultures

For the production of Gruyère cheese, the use of NWCs, where *L. helveticus* is a predominant species, is regulated. For quality management, therefore, determining the composition of NWCs at the species, and preferably at the strain level, is very relevant. To test whether the complete genomes of the three newly sequenced strains could contribute to a better assignment of the species composition of NWCs, and in particular, whether the genomes could help to distinguish different strains of a species (Smid et al., 2014), a metagenomic analysis of a Gruyère whey starter culture from a cheese factory in Switzerland was performed using the Illumina HiSeq platform.

**Figure 7:**
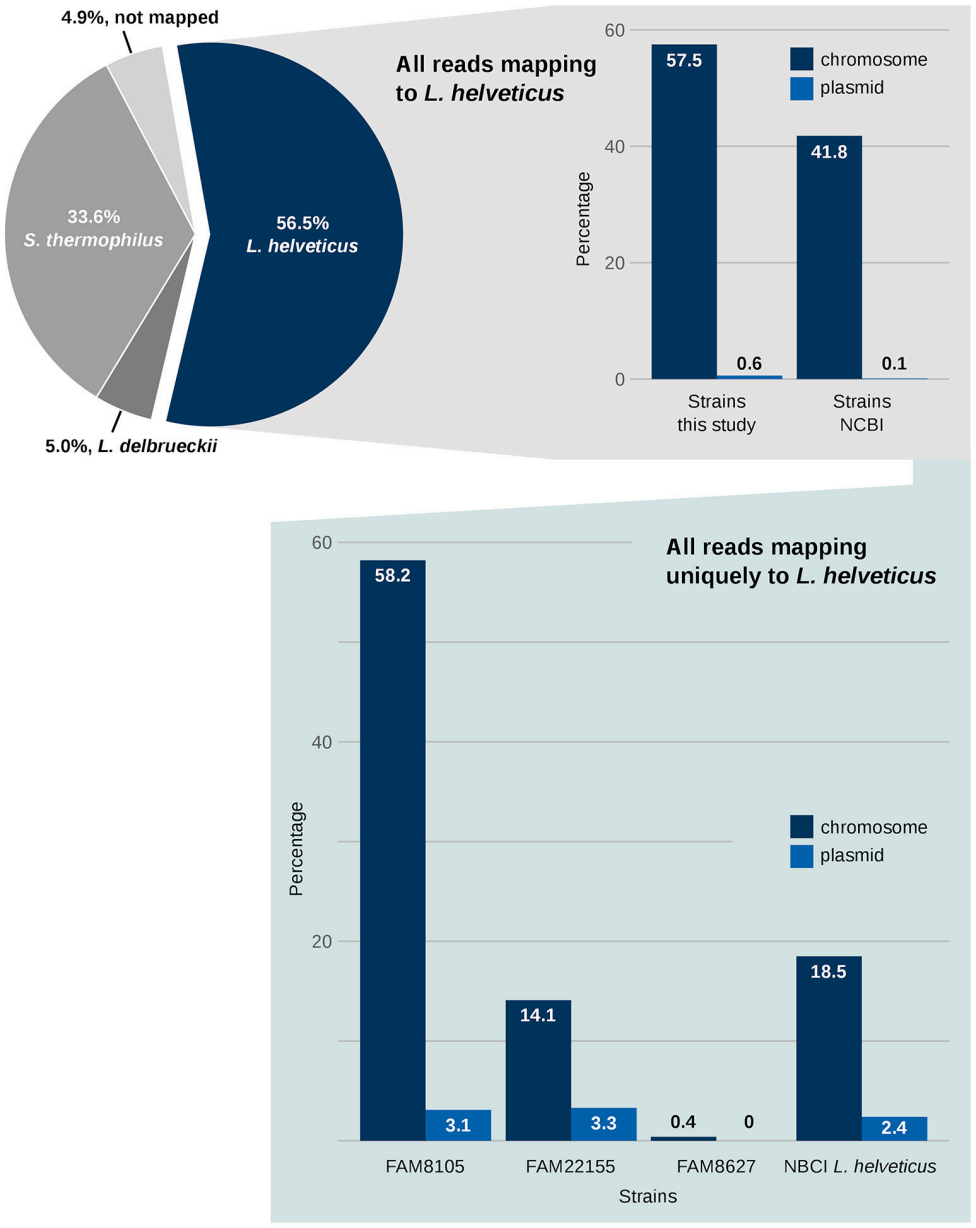
Metagenomics of NWCs benefits from the availability of complete genome data. The metagenomic analysis of a NWC reveals the qualitative species level composition (pie chart). A mapping of the reads against all 12 *L. helveticus* genomes provides a rough separation of reads that can map to several of the NCBI complete genomes vs. reads assigned to our three FAM strains (upper right panel; see Methods). A focus on sequences that uniquely map to only one *L. helveticus* strain is shown in the lower panel.

First, the species composition of the NWC was determined. Using a reference-based approach, the Illumina HiSeq reads were mapped to the genomes of the strains that were able to explain 95% of the reads of the metagenome sample (see Methods). About 57% of the reads originated from *L. helveticus*, 34% from *S. thermophilus* and 5% from *L. delbrueckii*, respectively (Figure 7, pie chart). Another 5% of the reads could not be mapped. However, this last percentage might possibly decrease if the—based on the mapped reads—“rare” species in the sample were also considered, which was beyond the scope of this analysis. The percentages from this whole genome sequencing-based metagenomics approach are comparable to those reported in a reverse transcriptase length heterogeneity PCR-based analysis of the composition of NWCs in Grana Padano (Rossetti et al., 2008), where the domain A of the variable 16S rRNA gene was assessed. *L. helveticus* strains were always dominant, while the percentages of *S. thermophilus* and *L. delbrueckii* seemed to vary in these cheese whey starters.

Next, the relevance of the three FAM strains in relation to existing NCBI RefSeq *L. helveticus* strains was assessed. To achieve this, the mapping information above was filtered for two groups: (1) reads mapping only to FAM strains and (2) reads mapping only to NCBI RefSeq strains (Figure 7, right upper panel). Although this information provides only a “semi-quantitative” picture, nevertheless, it emphasizes the relevance of the FAM strains for this NWC from a Gruyère cheese.

Finally, we determined the numbers of all uniquely mapped HiSeq reads, i.e., reads that mapped exclusively to one target sequence among the *L. helveticus* strains. The result of this analysis showed a high relevance of FAM8105 and FAM22155 compared to the RefSeq *L. helveticus* reference strains. In contrast, strain FAM8627 had virtually no unique mappings (Figure 7, right lower panel).

## Discussion

Since the introduction in 2009, the average read length of the PacBio third-generation NGS technology has been steadily increasing (Eid et al., 2009). Together with modern genome assembly algorithms (Koren et al., 2012; Chin et al., 2013), this technology is revolutionizing the ability to sequence microbial genomes and to subsequently study their function. Our data demonstrated that by using long read technologies, even repeat-rich class II genomes can readily be *de novo* assembled into complete and highly accurate genome sequences. The comparison of the MiSeq- and PacBio-based assemblies emphasized that existing strain differences can be overlooked, and that not only accessory genes but also core genes can be missed by the fragmented assemblies based on short reads. PacBio data, thus, are particularly suited to describe full genomes of LAB, which harbor a large number of repeats. Although more expensive, a complete genome sequence based on long read technologies represents an optimal basis for subsequent accurate and in-depth genome annotation (Omasits et al., 2017). Furthermore, complete genomes enable researchers to study genome rearrangements and evolution over time (Callanan et al., 2008), and provide the basis for using strain-specific sequences for diagnostic purposes (Ercolini, 2013), to create accurate, genome-scale metabolic models (Stefanovic et al., 2017), and to carry out functional genomics studies relying on condition-specific gene or protein expression data (Omasits et al., 2013).

Compared to a large phylogenomic profiling study of LAB (Sun et al., 2015) which included two *L. helveticus* strains, we provided a more detailed phylogeny of *L. helveticus* strains, which formed a distinct clade among LAB. Notably, two strains from different subclades (CNRZ 32 and FAM22155) lacked a detectable CRISPR/Cas system. An assessment of other LAB using pre-computed datasets from CRISPRfinder (http://crispr.i2bc.paris-saclay.fr/crispr/) indicated that this system was also lost in a few of the analyzed *L. acidophilus* and *L. delbrueckii* strains. In contrast, loss among *L. johnsonii* strains was more prominent, as three out of five (60%) lacked the system. Thus, the CRISPR/Cas system may not be as useful for strain typing as previously proposed (Selle and Barrangou, 2015). These strains have likely acquired other defense mechanisms against phage attack, which is quite frequent in the dairy environment (Samson and Moineau, 2013). Some of these mechanisms can likely be found among the enriched COG class of “defense mechanisms” (V) among accessory and unique genes (see below).

The results of the first pan-core genome analysis for *L. helveticus* based on 12 complete genomes indicated that the ratio of pan and core genes was comparable to that reported for *L. casei* (Broadbent et al., 2012). Although the core genome was enriched in genes of known function, 132 of the 988 core gene clusters (13.2%) were annotated as “hypothetical protein.” Particularly interesting was the enrichment of the functional COG categories “cell wall and membrane biogenesis” (M) among the unique genes and “defense mechanisms” (V) among the accessory and unique genes. The COG class “unknown function” (S) was also overrepresented among unique genes, indicating that significant effort will be required to unravel the putative functions carried out by the unique genes on top of the known role of surface-localized proteins. Furthermore, some of the strain-specific sequences included insertion elements (IE) and transposons (Supplementary Results in Supplementary Data Sheet 1), which could be exploited for diagnostic purposes.

The pseudogene analysis also supported the observation that the nutrient-rich conditions encountered by *L. helveticus* strains in their natural habitat favor the accumulation of repeats and insertion sequences and that their genomes are undergoing reductive genome evolution (Callanan et al., 2008; Broadbent et al., 2013). Consistent with this, it has been recently shown in *Escherichia coli* that gene loss in nutrient-rich environments can serve as a significant fitness advantage for auxotrophic mutants (D'Souza and Kost, 2016). Moreover, the pseudogene analysis indicated that genes for lipid metabolic processes were affected in all three FAM strains, in particular genes involved in isoprenoid biosynthesis. This observation can be explained as an adaptation to a low pH environment. During milk fermentation, the pH naturally drops due to lactic acid production. To counteract this stress factor, *L. helveticus* could have evolved to preferentially use acetyl-CoA for the biosynthesis of saturated fatty acids instead of isoprenoids to stabilize the cell membrane. This hypothesis is in line with a proteomic study by (Fernandez et al., 2008), who found that *L. delbrueckii* subsp. *bulgaricus* repressed enzymes involved in isoprenoid biosynthesis during acid stress.

Our analysis of amino acid metabolism genes based on KEGG pathways suggested that all strains are auxotrophs for at least 12 amino acids, which is in accordance with strain CNRZ 32 that was described to be auxotroph for 14 amino acids (Christiansen et al., 2008). Moreover, amino acid transport systems are often found among pseudogenes. This seems to be an evolutionary consequence of the low amount of free amino acids present in milk. The presence of oligopeptide transport systems and a broad peptidase complement in *L. helveticus* suggests that all essential amino acids are supplied by the internal breakdown of peptides in this species.

The genome mining effort provided direct evidence for a genotype to phenotype link for PrtH3, a member of the CEPs, which correlated with the biochemical analysis for strain FAM8627. The genome mining also suggested an indirect link for the PGH gene family that will require further experiments. The PGH complement and the cell wall composition, have been postulated to represent factors that contribute to different autolytic potential of *L. helveticus* strains (Jebava et al., 2011; Vinogradov et al., 2013). As Jebava et al. reported that 24 *L. helveticus* strains expressed all nine genes, differential gene expression does not seem to be related to different autolytic properties. The present data—in contrast to Jebava et al.'s PCR data—suggested several differences in the PGH complement among the strains, and only five of nine genes were present in the core genome. The remaining four genes were mutated in at least one of the strains likely resulting in at least partial loss of their function. These *in silico* analyses should thus ideally be further complemented by in-depth proteomics profiling experiments (Ahrens et al., 2010), such as a comprehensive analysis of the subcellular localization data of condition-specific proteomes including rich surface proteomes (Stekhoven et al., 2014), to explore a potential correlation between differential protein expression and varying autolytic properties. However, in line with the alternative hypothesis that the cell wall composition is a key factor for autolysis (Vinogradov et al., 2013), we observed that the COG category for “cell wall/membrane biogenesis” was enriched in the unique genes of *L. helveticus* strains indicating that cell wall composition may vary between strains. Thus, further studies on not only membrane proteins but also the chemical composition and the biochemical synthesis of the cell wall are needed to help unravel the molecular mechanisms of autolysis in *L. helveticus*.

The availability of more complete genomes is highly relevant to study the composition of metagenomes in more detail and beyond 16S rRNA analysis (De Filippis et al., 2014; Ellegaard and Engel, 2016). The present whole genome sequencing-based metagenome analysis of an NWC demonstrated that complete genome sequences can help to decipher the strain composition in moderately complex metagenomes (Erkus et al., 2013), such as those observed in raw milk or cheese starter cultures (Smid et al., 2014). Particularly promising is the potential to assemble the genomes of different strains directly from such moderately complex mixtures (Sangwan et al., 2016). Although this assembly will be challenging when closely related genomes are present in the mixture (Brown, 2015), complete genome information is one of the key factors to further exploit the exceptional potential of lactobacilli for various biotechnological applications.

## Data access

The genome sequences of the three *L. helveticus* strains are available from NCBI GenBank under accession numbers CP015496 & CP015497 (FAM8105), CP015498 & CP015499 (FAM22155), and CP015444 & CP015445 (FAM8627) (Table 1). Furthermore, raw sequence data (and methylation analysis) has been submitted to the NCBI Sequence Read Archive (SRA): SRX1725197 (FAM8105), SRX1726542 (FAM2155), SRX1726359 (FAM8607), see also Supplementary Table 1.

## Author contributions

DM, AV, and MS: assembled genomes, MS: carried out bioinformatic analyses, genome annotation, and created figures, JM: mined the genomes for genes of interest; AM: cultivated *L. helveticus* strains, extracted gDNA and created light microscopy images; AW: set up the SMRT portal and developed the repeat analysis web server together with MB; VS: explored differences between short read and PacBio based assemblies; CW: performed enzymatic assays; JF and EE-M: participated in study design and data interpretation; SI: oversaw the culturing, biochemical analyses, and selection of genes of interest; CA: conceived the study, oversaw bioinformatics analyses, repeat server functionality; MS and CA: wrote the paper.

### Conflict of interest statement

The authors declare that the research was conducted in the absence of any commercial or financial relationships that could be construed as a potential conflict of interest.

## Supplementary material

Supplementary Table 1 Metrics for the PacBio SMRT sequencing runs and corresponding NCBI Sequence Read Archive (SRA) accession numbers.

Supplementary Table 2 Genomic positions of predicted genomic islands and prophages.

Supplementary Table 3 Overview of bacterial strains used for phylogenetic analyses in this study.

Supplementary Table 4 IS elements identified in the three *L. helveticus* genomes. Additional to the analysis using TnpPred, the IS elements were also identified using ISfinder (https://www-is.biotoul.fr). For our three FAM strains, this resulted in more IS elements than the TnpPred analysis. Some of the IS elements were specific to one *L. helveticus* FAM strain and thus might be used for diagnostic applications. Pseudogenes which are attributed to IS sequences are noted in round brackets. IS sequences localized in plasmids are noted in square brackets (they only occurred in FAM8105).

Supplementary Table 5 CEP activity of *L. helveticus* FAM8105, FAM22155 and FAM8627.

Supplementary Table 6 Overview of predicted 6-phospho-beta-glucosidase gene products in complete *L. helveticus* genomes. Presence/absence table for five 6-phospho-beta-glucosidase CDSs detected in the twelve completely sequenced strains. Tick marks (✓) represent genes which are detected and predicted to be functional. “P” marks genes that were predicted as pseudogenes by the NCBI annotation. In the first row the accession number of a representative NCBI RefSeq protein is given for every group.

Supplementary Table 7 Overview of genes related to lipid metabolism for all complete *L. helveticus* genomes. Presence/absence table for four genes related to lipid metabolism detected either as intact or pseudogene in all completely sequenced strains. Tick marks (✓) represent genes which are detected and predicted to be functional. “P” marks genes that were predicted as pseudogenes by the NCBI annotation pipeline.

Supplementary Table 8 Analysis of the presence of peptide transporters, proteinases and peptidases in selected Lactobacillus strains. White, yellow and green table cells indicate absence, single and multiple genes, respectively.

Supplementary Table 9A *In silico* analysis of the amino acid biosynthetic capabilities of various Lactobacillus strains based on KEGG pathway annotation.

Supplementary Table 9B Details of *in silico* analysis for amino acid metabolism based on KEGG pathway.

Supplementary Table 10 Additional result files of the pan-core genome analysis of 12 *L. helveticus* genomes. We provide these files as a resource to the community; most files are text files (csv & faa) and are using Linux style formatted line breaks. In addition, we provide HMM profiles for core genome clusters.

Supplementary Figure 1 Light microscopic images of the three *L. helveticus* strains. **(A)** FAM8105, **(B)** FAM22155, and **(C)** FAM8627. In agreement with earlier reports, *L. helveticus* cells are predominantly rods or coccobacilli (Claesson et al., 2007).

Supplementary Figure 2 Circular maps for plasmids of FAM8105, FAM22155, and FAM8627. The plots were generated using CGview (Stothard and Wishart, 2005). For each subfigure **(A)** FAM8105, **(B)** FAM22155, and **(C)** FAM8627, the following features are shown (moving from the outermost track inwards): (1) CDS on forward strand colored according to COG category, (2) CDS (*black*) on forward strand, (3) black line representing genome sequence, (4) CDS (*black*) on reverse strand, (5) CDS on reverse strand colored according to COG category, (6) GC content (*black*), (7) positive and negative GC skew (*green* and *purple*, respectively) and (8) genome position in kbp.

Supplementary Figure 3 Barplot showing the distribution of core, accessory and unique gene clusters among the 12 *L. helveticus* strains. The y-axis shows the number of gene clusters for every category, the x-axis shows how many strains contribute to the respective clusters. On the leftmost position (“1”) the number of clusters with gene(s) from only one strain is shown (“Unique genome”). On the rightmost position (“12”) the same is shown for the core genome (genes present in all strains). Everything in between (“2” – “11”) corresponds to gene clusters of the accessory genome.

Supplementary Figure 4 Distribution of the occurrence of eleven insertion sequence (IS) families among 12 *L. helveticus* strains shown as a heatmap. The background color corresponds to the number of ISs detected using TnpPred for an IS family for the respective strain (reflecting log values used for clustering). Strains and IS are clustered (hierarchical clustering using average linkage and euclidean distance based on log values) and the dendrogram is shown on top for IS and on the left for the strains. For FAM8105, the ISs detected on the plasmid are shown in brackets. White boxes at the bottom and at the right show the total for IS families and strains, respectively. For the remaining eight families (IS1, IS1380, IS21, IS481, IS630, IS91, ISAs1, Tn3), no hits were observed.

Supplementary Data Sheet 1 Supplementary Material and Methods & Results.

Supplementary Data Sheet 2 Result files of pan-core genome analysis. For descriptions, see Supplementary Table 10.
