# Supplementary material for: Comparative Genomics of Completely Sequenced Lactobacillus helveticus Genomes Provides Insights into Strain-Specific Genes and Resolves Metagenomics Data Down to the Strain Level
Source: Front Microbiol. 2018 Jan 30;9:63. doi: 10.3389/fmicb.2018.00063 (PMC5797582; doi:10.3389/fmicb.2018.00063)
Supplement: Supplementary Table 1 — Metrics for the PacBio SMRT sequencing runs and corresponding NCBI Sequence Read Archive (SRA) accession numbers. [file Table1.DOCX]

Supplementary Material

Comparative genomics of completely sequenced *Lactobacillus helveticus* genomes provides insights into strain-specific genes and resolves metagenomics data down to the strain level

**Supplementary Table 1:** Metrics for the PacBio SMRT sequencing runs and corresponding NCBI Sequence Read Archive (SRA) accession numbers.

|  | **FAM8105** | **FAM22155** | **FAM8627** |
| --- | --- | --- | --- |
| **Number of SMRT cells** | 3 | 3 | 3 |
| **No. of subreads** | 116,269 | 85,822 | 40,890 |
| **Mean subread length** | 10,587 | 9,578 | 11,808 |
| **NCBI SRA accession*** | SRX1725197 | SRX1726542 | SRX1726359 |

* Raw data (PacBio) and methylation analysis. The base modification analysis was performed on the final assemblies with protocol *RS_Modification_and_Motif_Analysis.1* from *smrtanalysis_2.3.0.*
