# Supplementary material for: Comparative Genomics of Completely Sequenced Lactobacillus helveticus Genomes Provides Insights into Strain-Specific Genes and Resolves Metagenomics Data Down to the Strain Level
Source: Front Microbiol. 2018 Jan 30;9:63. doi: 10.3389/fmicb.2018.00063 (PMC5797582; doi:10.3389/fmicb.2018.00063)
Supplement: Supplementary Table 2 — Genomic positions of predicted genomic islands and prophages. [file Table2.DOCX]

Supplementary Material

Comparative genomics of completely sequenced *Lactobacillus helveticus* genomes provides insights into strain-specific genes and resolves metagenomics data down to the strain level

**Supplementary Table 2:** Genomic positions of predicted genomic islands and prophages.

| **FAM8105** | | | | **FAM8627** | | | | **FAM22155** | | | |
| --- | --- | --- | --- | --- | --- | --- | --- | --- | --- | --- | --- |
| Genomic Islands**^1^** | | Prophages**^2^** | | Genomic Islands**^1^** | | Prophages**^2^** | | Genomic Islands**^1^** | | Prophages**^2^** | |
| from | to | from | to | from | to | from | to | from | to | from | to |
| 50898 | 58899 | 490573 | 531557 | 130712 | 135091 | 1843584 | 1852582 | 63951 | 74079 | 1999713 | 2016697 |
| 51527 | 58003 | 1439648 | 1447030 | 143161 | 148138 |  |  | 66974 | 74975 |  |  |
| 178906 | 190440 | 1458832 | 1501450 | 690074 | 702417 |  |  | 256492 | 268315 |  |  |
| 288631 | 321231 | 2015994 | 2031360 | 1828304 | 1833766 |  |  | 317507 | 350106 |  |  |
| 1477807 | 1484274 |  |  | 1849031 | 1862275 |  |  | 1018897 | 1027497 |  |  |
| 1711758 | 1717678 |  |  | 1876223 | 1909494 |  |  | 1168077 | 1174648 |  |  |
| 1714863 | 1722511 |  |  | 1917652 | 1922362 |  |  | 1580123 | 1584598 |  |  |
| 1782724 | 1807113 |  |  |  |  |  |  | 1768266 | 1772908 |  |  |
| 1831931 | 1836844 |  |  |  |  |  |  | 1859862 | 1876069 |  |  |
| 1903244 | 1907257 |  |  |  |  |  |  | 1890361 | 1905131 |  |  |
| 1906286 | 1918803 |  |  |  |  |  |  | 1908933 | 1922065 |  |  |
| 1910197 | 1917682 |  |  |  |  |  |  | 2023130 | 2057094 |  |  |
| 2082386 | 2087304 |  |  |  |  |  |  | 2065148 | 2070066 |  |  |
|  |  |  |  |  |  |  |  | 2119059 | 2129118 |  |  |

^1^ Intervals were de-replicated from raw output of IslandViewer 3. Overlapping intervals were NOT collapsed and can be overlapping by large parts.

^2^ Type (Phaster classes)

| intact | incomplete | questionable |
| --- | --- | --- |
