# Supplementary material for: Comparative Genomics of Completely Sequenced Lactobacillus helveticus Genomes Provides Insights into Strain-Specific Genes and Resolves Metagenomics Data Down to the Strain Level
Source: Front Microbiol. 2018 Jan 30;9:63. doi: 10.3389/fmicb.2018.00063 (PMC5797582; doi:10.3389/fmicb.2018.00063)
Supplement: Supplementary Table 3 — Overview of bacterial strains used for phylogenetic analyses in this study. [file Table3.DOCX]

Supplementary Material

Comparative genomics of completely sequenced Lactobacillus helveticus genomes provides insights into strain-specific genes and resolves metagenomics data down to the strain level

**Supplementary Table 3:** Overview of bacterial strains used for phylogenetic analyses in this study.

| **Species** | **Strain** | **Accession** | **Comment** |
| --- | --- | --- | --- |
| *Bacillus subtilis subsp. subtilis* | Str. 168 | NC_000964 | Outgroup |
| *Enterococcus faecalis* | V583 | NC_004668 | LAB |
| *Leuconostoc mesenteroides subsp. mesenteroides* | ATCC 8293 | NC_008531 | LAB |
| *Oenococcus oeni* | PSU-1 | NC_008528 | LAB |
| *Pediococcus pentosaceus* | ATCC 25745 | NC_008525 | LAB |
| *Streptococcus thermophilus* | LMG 18311 | NC_006448 | LAB |
| *Lactobacillus acidophilus* | NCFM | NC_006814 | Genus *Lactobacillus* |
| *Lactobacillus brevis* | ATCC 367 | NC_008497 | Genus *Lactobacillus* |
| *Lactobacillus casei* | LOCK919 | NC_021721 | Genus *Lactobacillus* |
| *Lactobacillus delbrueckii subsp. bulgaricus* | ATCC 11842 | NC_008054 | Genus *Lactobacillus* |
| *Lactobacillus gasseri* | ATCC 33323 | NC_008530 | Genus *Lactobacillus* |
| *Lactobacillus johnsonii* | NCC 533 | NC_005362 | Genus *Lactobacillus* |
| *Lactobacillus plantarum* | WCFS1 | NC_004567 | Genus *Lactobacillus* |
| *Lactobacillus sakei subsp. sakei* | 23k | NC_007576 | Genus *Lactobacillus* |
| *Lactobacillus salivarius* | UCC118 | NC_007929 | Genus *Lactobacillus* |
| *Lactobacillus helveticus* | CAUH18 | NZ_CP012381 | *Lactobacillus helveticus* |
| *Lactobacillus helveticus* | CNRZ32 | NC_021744 | *Lactobacillus helveticus* |
| *Lactobacillus helveticus* | D76 | NZ_CP016827 | *Lactobacillus helveticus* |
| *Lactobacillus helveticus* | DPC 4571 | NC_010080 | *Lactobacillus helveticus* |
| *Lactobacillus helveticus* | H10 | NC_017467 | *Lactobacillus helveticus* |
| *Lactobacillus helveticus* | H9 | NZ_CP002427 | *Lactobacillus helveticus* |
| *Lactobacillus helveticus* | KLDS1.8701 | NZ_CP009907 | *Lactobacillus helveticus* |
| *Lactobacillus helveticus* | MB2-1 | NZ_CP011386 | *Lactobacillus helveticus* |
| *Lactobacillus helveticus* | R0052 | NC_018528 | *Lactobacillus helveticus* |
| *Lactobacillus helveticus* | FAM8105 | CP015496 | *L. helveticus* **(this publication)** |
| *Lactobacillus helveticus* | FAM22155 | CP015498 | *L. helveticus* (**this publication**) |
| *Lactobacillus helveticus* | FAM8627 | CP015444 | *L. helveticus* (**this publication**) |
