# Supplementary material for: Comparative Genomics of Completely Sequenced Lactobacillus helveticus Genomes Provides Insights into Strain-Specific Genes and Resolves Metagenomics Data Down to the Strain Level
Source: Front Microbiol. 2018 Jan 30;9:63. doi: 10.3389/fmicb.2018.00063 (PMC5797582; doi:10.3389/fmicb.2018.00063)
Supplement: Supplementary Table 4 — IS elements identified in the three L. helveticus genomes. Additional to the analysis using TnpPred, the IS elements were also identified using ISfinder (https://www-is.biotoul.fr). For our three FAM strains, this resulted in more IS elements than the TnpPred analysis. Some of the IS elements were specific to one L. helveticus FAM strain and thus might be used for diagnostic applications. Pseudogenes which are attributed to IS sequences are noted in round brackets. IS sequences localized in plasmids are noted in square brackets (they only occurred in FAM8105). [file Table4.DOCX]

Supplementary Material

Comparative genomics of completely sequenced *Lactobacillus helveticus* genomes provides insights into strain-specific genes and resolves metagenomics data down to the strain level

**Supplementary Table 4:** IS elements identified in the three *L. helveticus* genomes. Additional to the analysis using TnpPred, the IS elements were also identified using ISfinder (https://www-is.biotoul.fr). For our three FAM strains, this resulted in more IS elements than the TnpPred analysis. Some of the IS elements were specific to one *L. helveticus* FAM strain and thus might be used for diagnostic applications. Pseudogenes which are attributed to IS sequences are noted in round brackets. IS sequences localized in plasmids are noted in square brackets (they only occurred in FAM8105).

| **IS Name** | **Origin / first isolation *** | **IS Family *** | **FAM8105** | **FAM22155** | **FAM8627** |
| --- | --- | --- | --- | --- | --- |
| IS1070 | *Leuconostoc Lactis*  (Vaughan and de Vos, 1995) | IS30 (Mahillon and Chandler, 1998) | 0 | 0 | 1 |
| IS1165 | *Lactobacillus helveticus* and others  (Johansen and Kibenich, 1992) | ISL3 (Mahillon and Chandler, 1998) | 2 | 1 | 1 |
| IS1201 | *Lactobacillus helveticus*  [(Tailliez et al. 1994)](https://paperpile.com/c/IrdTlz/l6t6) | IS256 (Mahillon and Chandler, 1998) | 67 (6) [2] | 61 (6) | 18 (4) |
| IS153 | *Lactobacillus sanfranciscensis*  (Ehrmann and Vogel, 2001) | IS3 (see citation “origin”) | 0 | 1 | 0 |
| ISAcba1 | *Actinobacteria* | IS1595 | 3 | 3 | 1 |
| ISApl1 | *Actinobacillus pleuropneumoniae* | IS30 | 1 | 0 | 0 |
| ISCbo10 | *Clostridium botulinum* | IS607 | 10 | 9 | 9 |
| ISCce5 | *Clostridium cellulolyticum* | IS66 | 1 | 4 | 6 |
| ISL2 | *Lactobacillus helveticus* | IS5 | 2 (1) | 11 (1) | 1 |
| ISL2A | *Lactobacillus helveticus* | IS5 | 1 | 9 | 5 |
| ISL5 | *Lactobacillus delbrueckii* | IS4 | 0 | 0 | 1 (3) |
| ISL6 | *Lactobacillus delbrueckii* | IS3 | 6 [2] | 6 | 4 |
| ISLac1 | *Lactobacillus acidophilus* | IS1182 | 7 (3) | 7 (5) | 6 (5) |
| ISLde1 | *Lactobacillus delbrueckii* | ISLre2 | 8 | 7 | 2 (3) |
| ISLga1 | *Lactobacillus gasseri* | IS30 | 2 | 2 | 9 |
| ISLh1 | *Lactobacillus helveticus* | IS982 | 5 | 8 (1) | 14 (1) |
| ISLhe1 | *Lactobacillus helveticus* | IS982 | 0 (3) | 0 (4) | 1 |
| ISLhe10 | *Lactobacillus helveticus* | ISLre2 | 21 (5) | 9 (3) | 3 (2) |
| ISLhe11 | *Lactobacillus helveticus* | ISLre2 | 0 (4) | 0 (4) | 1 (3) |
| ISLhe13 | *Lactobacillus helveticus* | ISLre2 | 1 (2) | 0 (4) | 0 (1) |
| ISLhe2 | *Lactobacillus helveticus* | ISL3 | 32 (6) | 20 (8) | 8 (11) |
| ISLhe4 | *Lactobacillus helveticus* | IS110 | 3 (2) | 6 | 8 (2) |
| ISLhe5 | *Lactobacillus helveticus* | IS982 | 0 (2) | 1 (1) | 3 (1) |
| ISLhe6 | *Lactobacillus helveticus* | IS3 | 3 | 2 (1) | 5 |
| ISLhe65 | *Lactobacillus helveticus* | IS200/IS605 | 18 (15) | 20 (13) | 18 (11) |
| ISLhe7 | *Lactobacillus helveticus* | IS982 | 2 | 1 | 0 (2) |
| ISLhe9 | *Lactobacillus helveticus* | IS607 | 2 | 2 | 0 |
| ISLjo1 | *Lactobacillus johnsonii* | IS30 | 3 | 3 (2) | 1 (6) |
| ISLjo5 | *Lactobacillus johnsonii* | IS200/IS605 | 1 | 1 | 0 |
| ISLke1 | *Lactobacillus kefiranofaciens* | ISLre2 | 0 | 0 (2) | 0 |
| ISLpl1 | *Lactobacillus plantarum* | IS30 | 0 (1) | 0 (1) | 0 (1) |
| ISLre1 | *Lactobacillus reuteri* | IS4 | 1 | 1 | 1 |
| ISPp1 | *Pediococcus pentosaceus* | IS30 | 0 | 0 | 0 (1) |
| ISStma11 | *Stenotrophomonas maltophilia* | ISL3 | 1 | 0 | 0 |
| **Total** |  |  | **203 (50) [4]** | **195 (56)** | **127 (57)** |

* According to ISfinder website (https://www-is.biotoul.fr/list_names_attributed.php) where not stated otherwise

**References**

Ehrmann, M. A., and Vogel, R. E. (2001). Characterisation of IS153, an IS3-family insertion sequence isolated from Lactobacillus sanfranciscensis and its use for strain differentiation. *Syst. Appl. Microbiol.* 24, 443–450. Available at: https://www.ncbi.nlm.nih.gov/pubmed/11822682.

Johansen, E., and Kibenich, A. (1992). Isolation and characterization of IS1165, an insertion sequence of Leuconostoc mesenteroides subsp. cremoris and other lactic acid bacteria. *Plasmid* 27, 200–206. Available at: https://www.ncbi.nlm.nih.gov/pubmed/1325060.

Mahillon, J., and Chandler, M. (1998). Insertion sequences. *Microbiol. Mol. Biol. Rev.* 62, 725–774. Available at: https://www.ncbi.nlm.nih.gov/pubmed/9729608.

Tailliez, P., Ehrlich, S. D., and Chopin, M. C. (1994). Characterization of IS1201, an insertion sequence isolated from Lactobacillus helveticus. *Gene* 145, 75–79. Available at: https://www.ncbi.nlm.nih.gov/pubmed/8045427.

Vaughan, E. E., and de Vos, W. M. (1995). Identification and characterization of the insertion element IS1070 from Leuconostoc lactis NZ6009. *Gene* 155, 95–100. Available at: https://www.ncbi.nlm.nih.gov/pubmed/7698675.
