# Supplementary material for: Comparative Genomics of Completely Sequenced Lactobacillus helveticus Genomes Provides Insights into Strain-Specific Genes and Resolves Metagenomics Data Down to the Strain Level
Source: Front Microbiol. 2018 Jan 30;9:63. doi: 10.3389/fmicb.2018.00063 (PMC5797582; doi:10.3389/fmicb.2018.00063)
Supplement: Supplementary Table 5 — CEP activity of L. helveticus FAM8105, FAM22155 and FAM8627. [file Table5.DOCX]

Supplementary Material

Comparative genomics of completely sequenced *Lactobacillus helveticus* genomes provides insights into strain-specific genes and resolves metagenomics data down to the strain level

**Supplementary Table 5:** CEP activity of *L. helveticus* FAM8105, FAM22155 and FAM8627.

| **Enzyme assay** | **FAM8105** | **FAM22155** | **FAM8627** |
| --- | --- | --- | --- |
| CEP (Azocasein)^1^ | 0.07 | 0.07 | not detected |

^1^ nmol min^-1^ OD_600_^-1^
