# Supplementary material for: Comparative Genomics of Completely Sequenced Lactobacillus helveticus Genomes Provides Insights into Strain-Specific Genes and Resolves Metagenomics Data Down to the Strain Level
Source: Front Microbiol. 2018 Jan 30;9:63. doi: 10.3389/fmicb.2018.00063 (PMC5797582; doi:10.3389/fmicb.2018.00063)
Supplement: Supplementary Table 6 — Overview of predicted 6-phospho-beta-glucosidase gene products in complete L. helveticus genomes. Presence/absence table for five 6-phospho-beta-glucosidase CDSs detected in the twelve completely sequenced strains. Tick marks (✓) represent genes which are detected and predicted to be functional. “P” marks genes that were predicted as pseudogenes by the NCBI annotation. In the first row the accession number of a representative NCBI RefSeq protein is given for every group. [file Table6.DOCX]

Supplementary Material

Comparative genomics of completely sequenced *Lactobacillus helveticus* genomes provides insights into strain-specific genes and resolves metagenomics data down to the strain level

Supplementary Table 6: Overview of predicted 6-phospho-beta-glucosidase gene products in complete *L. helveticus* genomes. Presence/absence table for five 6-phospho-beta-glucosidase CDSs detected in the twelve completely sequenced strains. Tick marks (✓) represent genes which are detected and predicted to be functional. “P” marks genes that were predicted as pseudogenes by the NCBI annotation. In the first row the accession number of a representative NCBI RefSeq protein is given for every group.

| **Representative NCBI Protein RefSeq** | **WP_012211748** | **WP_003627210** | **WP_014918986** | **WP_014919095** | **WP_080668557** |
| --- | --- | --- | --- | --- | --- |
| **FAM8105** | ✓ (Lh8105_06545) | ✓ (_03060) | P (_06605) | P (_07755) | P (_06570) |
| **FAM22155** | ✓ (Lh22155_06635) | ✓ (_02955) | P (_06705) | P (_07445) | P (_06660) |
| **FAM8627** | P (Lh8627_03355) | ✓ (_06590) | P (_03295) | P (_02460) | P (_03330) |
| **CAUH18** | ✓ (ALV80_RS06680) | P^*2^ (No locus tag) | ✓ (_RS06745) | P (_RS07460) | P (_RS06705) |
| **CNRZ 32** | ✓ (LHE_RS16560) | ✓ (_RS20560) | P (_RS16500) | P (_RS15655) | P (_RS16535) |
| **D76** | ✓ (BCM45_RS01080) | ✓ (_RS04505) | Not detected | P (_RS00185) | P (_RS01050) |
| **DPC 4571** | ✓ (LHV_RS04715) | ✓ (_RS08215) | P (_RS04635) | P (_RS03880) | P (_RS04690) |
| **H10** | ✓ (LBHH_RS06565) | P^*2^ (No locus tag) | ✓ (_RS06625) | P (_RS07395) | P (_RS06580) |
| **H9** | ✓ (LBH_RS04140) | ✓^*1^ (No locus tag) | P (_RS04075) | P (_RS03350) | P (_RS04115) |
| **KLDS1.8701** | ✓ (HUO_RS07390) | ✓ (_RS03800) | P (_RS07450) | P (_RS08330) | P (_RS07415) |
| **MB2-1** | P (TU99_RS04640) | ✓ (_RS08855) | Not detected | P (_RS03765) | P (_RS04615) |
| **R0052** | Not detected | ✓ (_RS02875) | ✓ (_RS06535) | ✓ (_RS07245) | Not detected |
| **Length of predicted protein (AAs)** | 460^*3^ | 48^*3^ | 481^*3^ | 491^*3^ | 211^*4^ |

*^1^ Not annotated, but present in chromosomal sequence. Most likely functional.

*^2^ Not annotated, but present in sequence. Includes premature stop codon.

*^3^ Annotations covering the whole RefSeq sequence.

*^4^ Annotations not covering the whole length of the RefSeq protein (235 aa).
