# Supplementary material for: Comparative Genomics of Completely Sequenced Lactobacillus helveticus Genomes Provides Insights into Strain-Specific Genes and Resolves Metagenomics Data Down to the Strain Level
Source: Front Microbiol. 2018 Jan 30;9:63. doi: 10.3389/fmicb.2018.00063 (PMC5797582; doi:10.3389/fmicb.2018.00063)
Supplement: Supplementary Table 7 — Overview of genes related to lipid metabolism for all complete L. helveticus genomes. Presence/absence table for four genes related to lipid metabolism detected either as intact or pseudogene in all completely sequenced strains. Tick marks (✓) represent genes which are detected and predicted to be functional. “P” marks genes that were predicted as pseudogenes by the NCBI annotation pipeline. [file Table7.DOCX]

Supplementary Material

Comparative genomics of completely sequenced *Lactobacillus helveticus* genomes provides insights into strain-specific genes and resolves metagenomics data down to the strain level

Supplementary Table 7: Overview of genes related to lipid metabolism for all complete *L. helveticus* genomes. Presence/absence table for four genes related to lipid metabolism detected either as intact or pseudogene in all completely sequenced strains. Tick marks (✓) represent genes which are detected and predicted to be functional. “P” marks genes that were predicted as pseudogenes by the NCBI annotation pipeline.

| **Annotation (NCBI RefSeq)** | **Farnesyl pyrophosphate synthetase** | **1-deoxy-D-xylulose-5-phosphate synthase** | **Glycerophosphodiester phosphodiesterase** | **Glycerophosphodiester phosphodiesterase 2** |
| --- | --- | --- | --- | --- |
| **FAM8105** | P (Lh8105_02525) | P (Lh8105_01790) | P (Lh8105_08970) | P (Lh8105_02235) |
| **FAM22155** | P (Lh22155_02665) | P (Lh22155_01965) | ✓ (Lh22155_08645) | ✓ (Lh22155_02375) |
| **FAM8627** | P (Lh8627_06845) | P (Lh8627_07595) | P (Lh8627_01345) | P (Lh8627_07140) |
| **CAUH18** | ✓ (ALV80_RS02510) | P (ALV80_RS01825) | ✓ (ALV80_RS08640) | P (ALV80_RS02220) |
| **CNRZ 32** | P (LHE_RS20845) | P (LHE_RS21590) | ✓ (LHE_RS14400) | P (LHE_RS21135) |
| **D76** | P (BCM45_RS04815) | P (BCM45_RS05505) | ✓ (BCM45_RS09290) | ✓ (BCM45_RS05110) |
| **DPC 4571** | P (LHV_RS08510) | P (LHV_RS01850) | ✓ (LHV_RS02710) | ✓ (LHV_RS02230) |
| **H10** | ✓ (LBHH_RS02460) | ✓ (LBHH_RS01780) | ✓ (LBHH_RS08520) | P (LBHH_RS02175) |
| **H9** | P (LBH_1442) | P (LBH_RS01615) | ✓ (LBH_RS02280) | P (LBH_RS01990) |
| **KLDS1.8701** | P (HUO_RS03545) | P (HUO_RS02795) | ✓ (HUO_RS09475) | P (HUO_RS03250) |
| **MB2-1** | P (TU99_RS09205) | P (TU99_RS01680) | ✓ (TU99_RS02620) | P (TU99_RS02145) |
| **R0052** | ✓ (R0052_RS02580) | ✓ (R0052_RS01750) | ✓ (R0052_RS08360) | ✓ (R0052_RS02295) |
| **Length of intact proteins (AAs)** | 326 | 580 | 231 | 456 |
