# Supplementary material for: Comparative Genomics of Completely Sequenced Lactobacillus helveticus Genomes Provides Insights into Strain-Specific Genes and Resolves Metagenomics Data Down to the Strain Level
Source: Front Microbiol. 2018 Jan 30;9:63. doi: 10.3389/fmicb.2018.00063 (PMC5797582; doi:10.3389/fmicb.2018.00063)
Supplement: Supplementary Table 8 — Analysis of the presence of peptide transporters, proteinases and peptidases in selected Lactobacillus strains. White, yellow and green table cells indicate absence, single and multiple genes, respectively. [file Table8.DOCX]

Supplementary Material

Comparative genomics of completely sequenced *Lactobacillus helveticus* genomes provides insights into strain-specific genes and resolves metagenomics data down to the strain level

**Supplementary Table 8:** Analysis of the presence of peptide transporters, proteinases and peptidases in selected *Lactobacillus* strains. White, yellow and green table cells indicate absence, single and multiple genes, respectively.

| **TRANSPORTERS** |  | *L. helv.* FAM 8105 | *L. helv.*  FAM  22155 | *L. helv.* FAM  8627 | *L. helv.* DPC 4571 | *L. helv.* CNRZ  32 | *L. helv.* H10 | *L. helv.* R0052 | *L. acid.* NCFM |
| --- | --- | --- | --- | --- | --- | --- | --- | --- | --- |
| ABC transporters  (oligopept.) | OppA | 1 | 1 | 1 | 1 | 1 | 1 | 1 | 3 |
|  | OppB | 1 | 1 | 1 | 1 | 1 | 1 | 1 | 1 |
|  | OppC | 1 | 1 | 1 | 1 | 1 | 1 | 1 | 1 |
|  | OppD | 1 | 1 | 1 | 1 | 1 | 1 | 1 | 1 |
|  | OppF | 1 | 1 | 1 | 1 | 1 | 1 | 1 | 1 |
| ABC transporters  (di/tri peptides) | DppA/P | 0 | 1 | 1 | 1 | 1 | 0 | 1 | 3 |
|  | DppB | 0 | 1 | 1 | 1 | 1 | 0 | 1 | 1 |
|  | DppC | 0 | 1 | 1 | 1 | 1 | 0 | 1 | 1 |
|  | DppD | 0 | 1 | 1 | 1 | 1 | 0 | 1 | 1 |
|  | DppF | 0 | 1 | 1 | 1 | 1 | 0 | 1 | 1 |
| Ion-linked  (di/tri peptides) | DtpT | **P** | **P** | **P** | **P** | **P** | 1 | 1 | 1 |
| P: Pseudogene |  |  |  |  |  |  |  |  |  |

| **PROTEINASES** |  | *L. helv.* FAM  8105 | *L. helv.* FAM 22155 | *L. helv.* FAM 8627 | *L. helv.* DPC 4571 | *L. helv.* CNRZ 32 | *L. helv.* H10 | *L. helv.* R0052 | *L. acid.* NCFM |
| --- | --- | --- | --- | --- | --- | --- | --- | --- | --- |
| cell envelope bound proteinases (CEPs) | PrtH1 | 0 | 0 | 1 | 0 | 1 | 0 | 0 | 0 |
|  | PrtH2 | 0 | 0 | 0 | 0 | 1 | 0 | 0 | 0 |
|  | PrtH3 | 1 | 1 | **P*** | **P#** | 1 | 0 | 0 | 0 |
|  | PrtH4 | 0 | 0 | 1 | 0 | 1 | 0 | 1 | 0 |
|  | PrtP | 0 | 0 | 0 | 0 | 0 | 1 | 0 | 1 |
| P: Pseudogene  * PrtH3 of FAM8627: frameshift after 185 aa; thus classified as pseudogene. (see Figure 6A, main text)  # PrtH3 of DPC 4571: stop codon close to C-terminus but likely still active since N-terminal peptidase domain is intact (see Figure 6A, main text) | | | | | | | | | |
| **PEPTIDASES** |  | *L. helv.* FAM 8105 | *L. helv.* FAM 22155 | *L. helv.* FAM 8627 | *L. helv.* DPC 4571 | *L. helv.* CNRZ 32 | *L. helv.* H10 | *L. helv.* R0052 | *L. acid.* NCFM |
| aminopeptidases | PepC | 1 | 1 | 1 | 1 | **P** | 1 | 1 | 1 |
|  | PepN | 1 | 1 | 1 | 1 | 1 | 1 | 1 | 1 |
|  | pepM | 1 | 1 | 1 | 1 | 1 | 1 | 1 | 1 |
|  | pepA | 1 | 1 | 1 | 1 | 1 | 1 | 1 | 1 |
|  | Pcp | 1 | 1 | 1 | 1 | 1 | 1 | 1 | 1 |
| endopeptidases | PepE | 2 **(1P)** | 3 | 3 | 3 | 3 | 3 | 3 | 3 |
|  | PepO | 3 | 3 | 3 | 3 | 3 | 2 **(1P)** | 3 | 2 |
|  | PepF | 1 | 1 | 1 | 1 | 1 | 1 | 1 | 1 |
| dipeptidases | PepD | 4 | 4 | 4 | 4 | 4 | 4 | 4 | 4 |
|  | PepV | 1 | 1 | 1 | 1 | 1 | 1 | 1 | 1 |
| tripeptidases | pepT | 2 | 2 | 2 | 2 | 2 | 2 | 2 | 2 |
| proline peptidases | PepX | 1 | 1 | 1 | 1 | 1 | 1 | 1 | 1 |
|  | PepI | 1 | 1 | 1 | 1 | 1 | 1 | 1 | 1 |
|  | PepR | 1 | 1 | 1 | 1 | 1 | 1 | 1 | 1 |
|  | PepL | 0 | 0 | 0 | 0 | 0 | 0 | 0 | 1 |
|  | PepP | 1 | 1 | 1 | 1 | 1 | 1 | 1 | 1 |
|  | PepQ | 1 | 1 | 1 | 1 | 1 | 1 | 1 | 1 |

P: Pseudogene
