# Supplementary material for: Comparative Genomics of Completely Sequenced Lactobacillus helveticus Genomes Provides Insights into Strain-Specific Genes and Resolves Metagenomics Data Down to the Strain Level
Source: Front Microbiol. 2018 Jan 30;9:63. doi: 10.3389/fmicb.2018.00063 (PMC5797582; doi:10.3389/fmicb.2018.00063)
Supplement: Supplementary Table 9A — In silico analysis of the amino acid biosynthetic capabilities of various Lactobacillus strains based on KEGG pathway annotation. [file Table9.DOCX]

Supplementary Material

Comparative genomics of completely sequenced *Lactobacillus helveticus* genomes provides insights into strain-specific genes and resolves metagenomics data down to the strain level

**Supplementary Table 9A:** *In silico* analysis of the amino acid biosynthetic capabilities of various *Lactobacillus* strains based on KEGG pathway annotation.

| **AA** | ***L. helv.***  ***FAM8105*** | ***L. helv.***  ***FAM22155*** | ***L. helv.***  ***FAM8627*** | ***L. helv.***  ***DPC 4571*** | ***L. helv.***  ***CNRZ 32*** | ***L. helv.***  ***H10*** | ***L. helv.***  ***R0052*** | ***L. acidophilus***  ***NCFM*** |
| --- | --- | --- | --- | --- | --- | --- | --- | --- |
| **Ser** | + | + | - | + | + | - | - | - |
| **Cys** | + | + | - | + | + | - | - | - |
| **Met** | - | - | - | - | - | - | - | - |
| **Ile** | - | - | - | - | - | - | - | - |
| **Leu** | - | - | - | - | - | - | - | - |
| **Val** | - | - | - | - | - | - | - | - |
| **Phe** | - | - | - | - | - | - | - | - |
| **Tyr** | - | - | - | - | - | - | - | - |
| **Trp** | - | - | - | - | - | - | - | - |
| **His** | - | - | - | - | - | - | - | - |
| **Pro** | - | - | - | - | - | - | - | - |
| **Gly** | + | + | From Ser | + | + | From Ser | From Ser | From Ser |
| **Thr** | - | - | - | - | - | From Asp | - | From Asp |
| **Asp** | From Asn | From Asn | From Asn | From Asn | From Asn | From Asn | From Asn | From Asn |
| **Asn** | From Asp | From Asp | From Asp | From Asp | From Asp | From Asp | From Asp | From Asp |
| **Lys** | From Asp | From Asp | From Asp | From Asp | From Asp | From Asp | From Asp | From Asp |
| **Ala** | - | - | - | - | - | From Asp | - | From Asp |
| **Arg** | - | - | - | - | - | - | - | - |
| **Glu** | - | - | - | - | - | - | - | - |
| **Gln** | From Glu | From Glu | From Glu | From Glu | From Glu | From Glu | From Glu | From Glu |

A “+” or “-“ symbol indicates that the considered amino acid (left column) can be *de novo* synthesized or not, respectively. The term “from x” indicates that a given amino acid (left column; e.g. Glycine) could be generated from another amino acid (in this case Serine) if present in the medium.

**Supplementary Table 9B:** Details of *in silico* analysis for amino acid metabolism based on KEGG pathway.

| **Amino acid(s)** | **Enzyme** | | **EC number** | **FAM8627** | **FAM22155** | **FAM8105** | **DPC 4571** | **CNRZ 32** | **H10** | **R0052** | **NCFM** |  |
| --- | --- | --- | --- | --- | --- | --- | --- | --- | --- | --- | --- | --- |
| **Ser** | Phosphoglycerate dehydrogenase | | EC 1.1.1.95 | No | Yes | Yes | Yes | Yes | No | No | No |  |
|  | Phosphoserine transaminase | | EC 2.6.1.52 | No | Yes | Yes | Yes | Yes | No | No | No |  |
|  | Phosphoserine phosphatase | | EC 3.1.3.3 | Yes | Yes | Yes | Yes | Yes | Yes | Yes | Yes |  |
|  | Glycine hydroxymethyltransferase | | EC 2.1.2.1 | Yes | Yes | Yes | Yes | Yes | Yes | Yes | Yes |  |
|  |  | |  |  |  |  |  |  |  |  |  |  |
|  |  | |  | **From Gly** | **Complete** | **Complete** | **Complete** | **Complete** | **From Gly** | **From Gly** | **From Gly** | **pathway** |
|  |  | |  |  |  |  |  |  |  |  |  |  |
| **Cys** | Serine acetyl-transferase | | EC 2.3.1.30 | Yes | Yes | Yes | Yes | Yes | No | Yes | No |  |
|  | *O*-Acetylserine (thiol) lyase | | EC 2.5.1.47 | Yes | Yes | Yes | Yes | Yes | Yes | Yes | Yes |  |
|  |  | |  |  |  |  |  |  |  |  |  |  |
|  |  | |  | **From Ser** | **Complete** | **Complete** | **Complete** | **Complete** | **Incomplete** | **From Ser** | **Incomplete** | **pathway** |
|  |  | |  |  |  |  |  |  |  |  |  |  |
| **Met** | Cystathionine beta-lyase | | EC 4.4.1.8 | Yes | Yes | Yes | Yes | Yes | Yes | Yes | Yes |  |
|  | 5-methyltetrahydropteroyltriglutamate-homocysteine S-methyltransferase | | EC 2.1.1.13 | Yes | Yes | Yes | Yes | Yes | Yes | Yes | No |  |
|  |  | |  |  |  |  |  |  |  |  |  |  |
|  |  | |  | **Incomplete** | **Incomplete** | **Incomplete** | **Incomplete** | **Incomplete** | **Incomplete** | **Incomplete** | **Incomplete** | **pathway** |
|  |  | |  |  |  |  |  |  |  |  |  |  |
| **Ile, Leu, Val** | Branched-chain aminotransferase | | EC 2.6.1.42 | Yes | Yes | Yes | Yes | Yes | Yes | Yes | Yes |  |
|  | Acetolactate synthase | | EC 2.2.1.6 | Yes | No | No | No | Yes | Yes | No | Yes |  |
|  |  | |  |  |  |  |  |  |  |  |  |  |
|  |  | |  | **Incomplete** | **Incomplete** | **Incomplete** | **Incomplete** | **Incomplete** | **Incomplete** | **Incomplete** | **Incomplete** | **pathway** |
|  |  | |  |  |  |  |  |  |  |  |  |  |
| **Phe, Tyr, Trp** | 3-Dehydroquinate dehydratase | | EC 4.2.1.10 | Yes | Yes*, but deleted | Yes*, but deleted | Yes | Yes | No | No | No | * 30% of the gene is deleted |
|  | Shikimate dehydrogenase | | EC 1.1.1.25 | Yes*, but deleted | Yes | Yes | Yes | Yes | No | No | No | * 19% of the gene is deleted |
|  | Anthranilate phosphoribosyltransferase | | EC 2.4.2.18 | No | No | No | No | No | No | Yes | No |  |
|  | Phosphoribosylanthranilate isomerase | | EC 5.3.1.24 | No | No | No | No | No | No | Yes | No |  |
|  | Indole-3-glycerol phosphate synthase | | EC 4.1.1.48 | No | No | No | No | No | No | Yes | No |  |
|  | Tryptophan synthase beta chain | | EC 4.2.1.20 | No | No | No | No | No | No | Yes | No |  |
|  | Aromatic aminotransferase | | EC 2.6.1.57 | Yes | Yes | Yes | Yes | Yes | Yes | Yes | Yes |  |
|  |  | |  |  |  |  |  |  |  |  |  |  |
|  |  | |  | **Incomplete** | **Incomplete** | **Incomplete** | **Incomplete** | **Incomplete** | **Incomplete** | **Incomplete** | **Incomplete** | **pathway** |
|  |  | |  |  |  |  |  |  |  |  |  |  |
| **His** | none | |  |  |  |  |  |  |  |  |  |  |
|  |  | |  |  |  |  |  |  |  |  |  |  |
| **Pro** | Pyrroline-5-carboxylate reductase | | EC 1.5.1.2 | No | No | No | No | No | No | No | Yes |  |
|  |  | |  |  |  |  |  |  |  |  |  |  |
|  |  | |  | **Incomplete** | **Incomplete** | **Incomplete** | **Incomplete** | **Incomplete** | **Incomplete** | **Incomplete** | **Incomplete** | **pathway** |
|  |  | |  |  |  |  |  |  |  |  |  |  |
| **Gly** | Glycine hydroxymethyltransferase | | EC 2.1.2.1 | Yes | Yes | Yes | Yes | Yes | Yes | Yes | Yes |  |
|  |  | |  |  |  |  |  |  |  |  |  |  |
|  |  | |  | **From Ser** | **Complete** | **Complete** | **Complete** | **Complete** | **From Ser** | **From Ser** | **From Ser** | **pathway** |
|  |  | |  |  |  |  |  |  |  |  |  |  |
|  | |  |  |  |  |  |  |  |  |  |  |  |
|  | |  |  |  |  |  |  |  |  |  |  |  |
| **Thr** | Aspartate kinase | | EC 2.7.2.4 | Yes | Yes | Yes | Yes | Yes | Yes | Yes | Yes |  |
|  | Aspartate-semialdehyde dehydrogenase | | EC 1.2.1.11 | Yes | Yes | Yes | Yes | Yes | Yes | Yes | Yes |  |
|  | Homoserine dehydrogenase | | EC 1.1.1.3 | No | No | No | No | No | Yes | Yes | Yes |  |
|  | Homoserine kinase | | EC 2.7.1.39 | No | No | No | No | No | Yes | No | Yes |  |
|  | Threonine synthase | | EC 4.2.3.1 | No | No | No | No | No | Yes | Yes | Yes |  |
|  |  | |  |  |  |  |  |  |  |  |  |  |
|  |  | |  | **Incomplete** | **Incomplete** | **Incomplete** | **Incomplete** | **Incomplete** | **From Asp** | **Incomplete** | **From Asp** | **pathway** |
|  |  | |  |  |  |  |  |  |  |  |  |  |
| **Asp** | Aspartate aminotransferase | | EC 2.6.1.1 | No* | No* | No* | Yes | Yes | Yes | Yes | Yes | * mutation(s) leads to multiple,shorter CDS |
|  |  | |  |  |  |  |  |  |  |  |  |  |
|  |  | |  | **Incomplete** | **Incomplete** | **Incomplete** | **Incomplete** | **Incomplete** | **Incomplete** | **Incomplete** | **Incomplete** |  |
|  |  | |  |  |  |  |  |  |  |  |  |  |
| **Asn** | Asparagine synthetase | | EC 6.3.1.1 | Yes | Yes | Yes | Yes | Yes | Yes | Yes | Yes |  |
|  |  | |  |  |  |  |  |  |  |  |  |  |
|  |  | |  | **From Asp** | **From Asp** | **From Asp** | **From Asp** | **From Asp** | **From Asp** | **From Asp** | **From Asp** |  |
|  |  | |  |  |  |  |  |  |  |  |  |  |
| **Lys** | Aspartate kinase | | EC 2.7.2.4 | Yes | Yes | Yes | Yes | Yes | Yes | Yes | Yes |  |
|  | Aspartate-semialdehyde dehydrogenase | | EC 1.2.1.11 | Yes | Yes | Yes | Yes | Yes | Yes | Yes | Yes |  |
|  | 4-Hydroxy-tetrahydrodipicolinate synthase | | EC 4.3.3.7 | Yes | Yes | Yes | Yes | Yes | Yes | Yes | Yes |  |
|  | 4-Hydroxy-tetrahydrodipicolinate reductase | | EC 1.17.1.8 | Yes | Yes | Yes | Yes | Yes | Yes | Yes | Yes |  |
|  | Tetrahydrodipicolinate N-acteyltransferase | | EC 2.3.1.89 | Yes | Yes | Yes | Yes | Yes | Yes | Yes | Yes |  |
|  | Aminotrasnferase | | EC 2.6.1.- | Yes | Yes | Yes | Yes | Yes | Yes | Yes | Yes |  |
|  | N-acetyldiaminopimelate deacetylase | | EC 3.5.1.47 | Yes | Yes | Yes | Yes | Yes | Yes | Yes | Yes |  |
|  | Diaminopimelate epimerase | | EC 5.1.1.7 | Yes | Yes | Yes | Yes | Yes | Yes | Yes | Yes |  |
|  | Diaminopimelate decarboxylase | | EC 4.1.1.20 | Yes | Yes | Yes | Yes | Yes | Yes | Yes | Yes |  |
|  |  | |  |  |  |  |  |  |  |  |  |  |
|  |  | |  | **From Asp** | **From Asp** | **From Asp** | **From Asp** | **From Asp** | **From Asp** | **From Asp** | **From Asp** |  |
|  |  | |  |  |  |  |  |  |  |  |  |  |
| **Ala** | Aspartate 4-decarboxylase | | EC 4.1.1.12 | No | No | No | No | No | Yes | No | Yes |  |
|  |  | |  |  |  |  |  |  |  |  |  |  |
|  |  | |  | **Incomplete** | **Incomplete** | **Incomplete** | **Incomplete** | **Incomplete** | **From Asp** | **Incomplete** | **From Asp** |  |
|  |  | |  |  |  |  |  |  |  |  |  |  |
| **Arg** | Ornithine carbamoyltransferase | | EC 2.1.3.3 | Yes*, but deleted | Yes*, but deleted | Yes*, but deleted | Yes | Yes | Yes | Yes | No | *strain 1: 42% is deleted. Strain2&3: 24% is deleted |
|  | Carbamoyl-phosphate synthase (large sub.) | | EC 6.3.5.5 | Yes | Yes | Yes | Yes | Yes | Yes | Yes | Yes |  |
|  | Carbamoyl-phosphate synthase (small sub.) | | EC 6.3.5.5 | No* | Yes | Yes | Yes | Yes | Yes | Yes | Yes | * mutation(s) leads to multiple,shorter CDS |
|  |  | |  |  |  |  |  |  |  |  |  |  |
|  |  | |  | **Incomplete** | **Incomplete** | **Incomplete** | **Incomplete** | **Incomplete** | **Incomplete** | **Incomplete** | **Incomplete** |  |
|  |  | |  |  |  |  |  |  |  |  |  |  |
|  |  | |  |  |  |  |  |  |  |  |  |  |
|  |  | |  |  |  |  |  |  |  |  |  |  |
|  |  | |  |  |  |  |  |  |  |  |  |  |
| **Glu** | Aspartate aminotransferase | | EC 2.6.1.1 | No* | No* | No* | Yes | Yes | Yes | Yes | Yes | * mutation(s) leads to multiple,shorter CDS |
|  |  | |  |  |  |  |  |  |  |  |  |  |
|  |  | |  | **Incomplete** | **Incomplete** | **Incomplete** | **Incomplete** | **Incomplete** | **Incomplete** | **Incomplete** | **Incomplete** |  |
|  |  | |  |  |  |  |  |  |  |  |  |  |
|  |  | |  |  |  |  |  |  |  |  |  |  |
| **Gln** | Glutamate-ammonia ligase | | EC 6.3.1.2 | Yes | Yes | Yes | Yes | Yes | Yes | Yes | Yes |  |
|  |  | |  |  |  |  |  |  |  |  |  |  |
|  |  | |  | **From Glu** | **From Glu** | **From Glu** | **From Glu** | **From Glu** | **From Glu** | **From Glu** | **From Glu** |  |
