# Supplementary material for: Comparative Genomics of Completely Sequenced Lactobacillus helveticus Genomes Provides Insights into Strain-Specific Genes and Resolves Metagenomics Data Down to the Strain Level
Source: Front Microbiol. 2018 Jan 30;9:63. doi: 10.3389/fmicb.2018.00063 (PMC5797582; doi:10.3389/fmicb.2018.00063)
Supplement: Supplementary Table 10 — Additional result files of the pan-core genome analysis of 12 L. helveticus genomes. We provide these files as a resource to the community; most files are text files (csv & faa) and are using Linux style formatted line breaks. In addition, we provide HMM profiles for core genome clusters. [file Table10.DOCX]

Supplementary Material

Comparative genomics of completely sequenced *Lactobacillus helveticus* genomes provides insights into strain-specific genes and resolves metagenomics data down to the strain level

**Supplementary Table 10:** Additional result files of the pan-core genome analysis of 12 *L. helveticus* genomes. We provide these files as a resource to the community; most files are text files (csv & faa) and are using Linux style formatted line breaks. In addition we provide HMM profiles for core genome clusters.

| **Gene lists of orthologous clusters (*.csv files)** | |
| --- | --- |
| Original output from Roary including all three stages (core, accessory and unique genome) | Lhelv_roary_raw_gene_presence_absence.csv |
| Pan genome clusters (I.e. all clusters) | Lhelv_pan_genome_clusters.csv |
| Core genome clusters | Lhelv_core_genome_clusters.csv |
| Accessory genome clusters | Lhelv_accessory_genome_clusters.csv |
| Unique genes per strain | Lhelv_[*strainname*]_unique_genes.csv |
| **Amino acid sequences for CDS of all twelve complete strains** | |
| Amino acid sequences for CDS of all twelve complete strains (including plasmids) | Lhelv_all_CDS.faa |
| **Representative amino acid sequences for gene clusters (*.faa files)** | |
| Representative sequences for core genome clusters | Lhelv_core_genome_representative.faa |
| Representative sequences for accessory genome clusters | Lhelv_accessory_genome_representative.faa |
| Sequences of unique genes per strain | Lhelv_[*strainname]*_unique_genes.faa |
| **HMM-models of core genome clusters** | |
| Profile HMMs for all core genome clusters (including one *.faa file and one MUSCLE alignment file per cluster) | Lhelv_core_genome_clusters_hmm.zip |
