# Supplementary figures and images for: Comparative Genomics of Completely Sequenced Lactobacillus helveticus Genomes Provides Insights into Strain-Specific Genes and Resolves Metagenomics Data Down to the Strain Level
Source: Front Microbiol. 2018 Jan 30;9:63. doi: 10.3389/fmicb.2018.00063 (PMC5797582; doi:10.3389/fmicb.2018.00063)

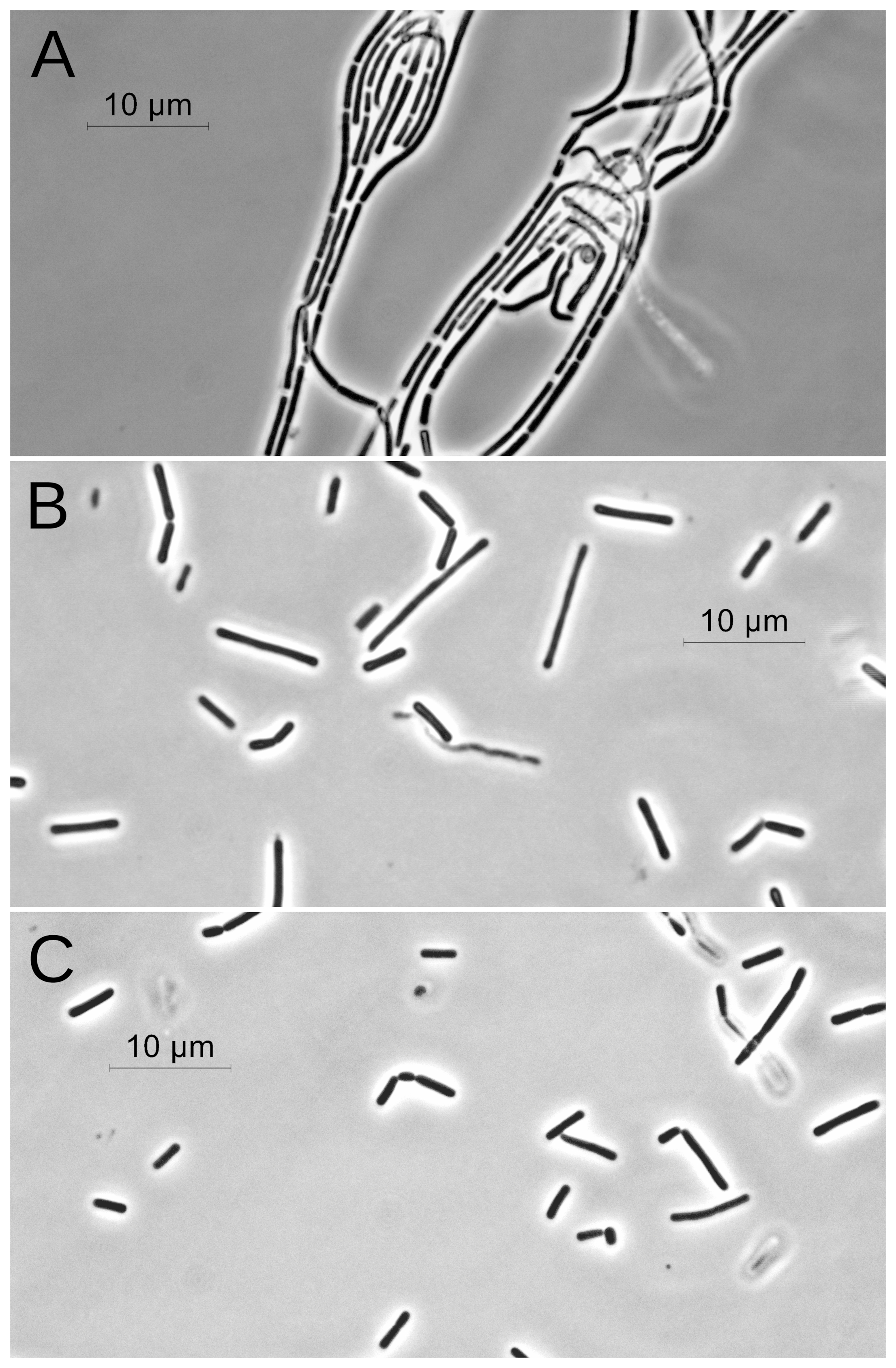

Supplement: Supplementary Figure 1 — Light microscopic images of the three L. helveticus strains. (A) FAM8105, (B) FAM22155, and (C) FAM8627. In agreement with earlier reports, L. helveticus cells are predominantly rods or coccobacilli (Claesson et al., 2007). [file Image1.JPEG]

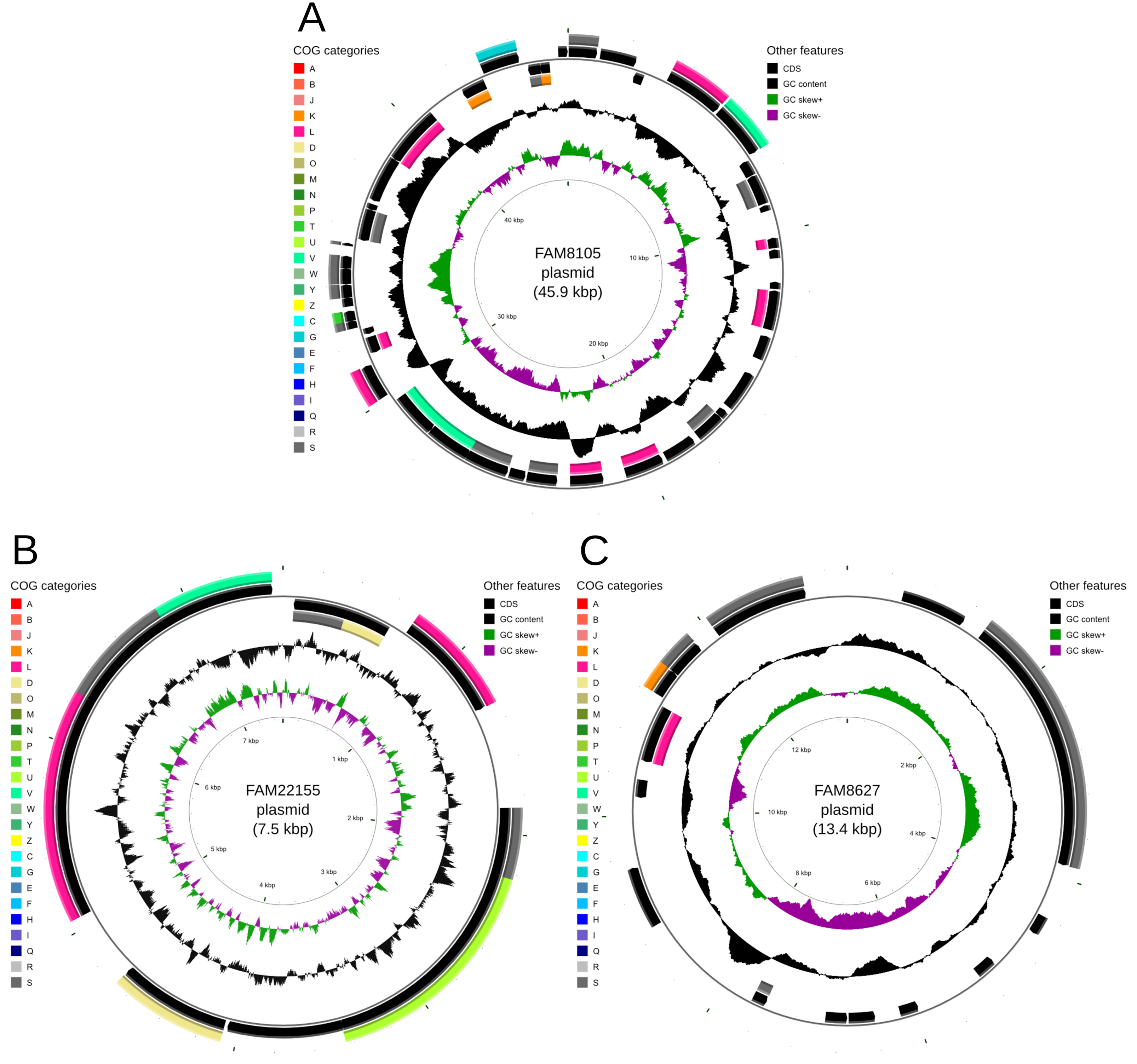

Supplement: Supplementary Figure 2 — Circular maps for plasmids of FAM8105, FAM22155, and FAM8627. The plots were generated using CGview (Stothard and Wishart, 2005). For each subfigure (A) FAM8105, (B) FAM22155, and (C) FAM8627, the following features are shown (moving from the outermost track inwards): (1) CDS on forward strand colored according to COG category, (2) CDS (black) on forward strand, (3) black line representing genome sequence, (4) CDS (black) on reverse strand, (5) CDS on reverse strand colored according to COG category, (6) GC content (black), (7) positive and negative GC skew (green and purple, respectively) and (8) genome position in kbp. [file Image2.JPEG]

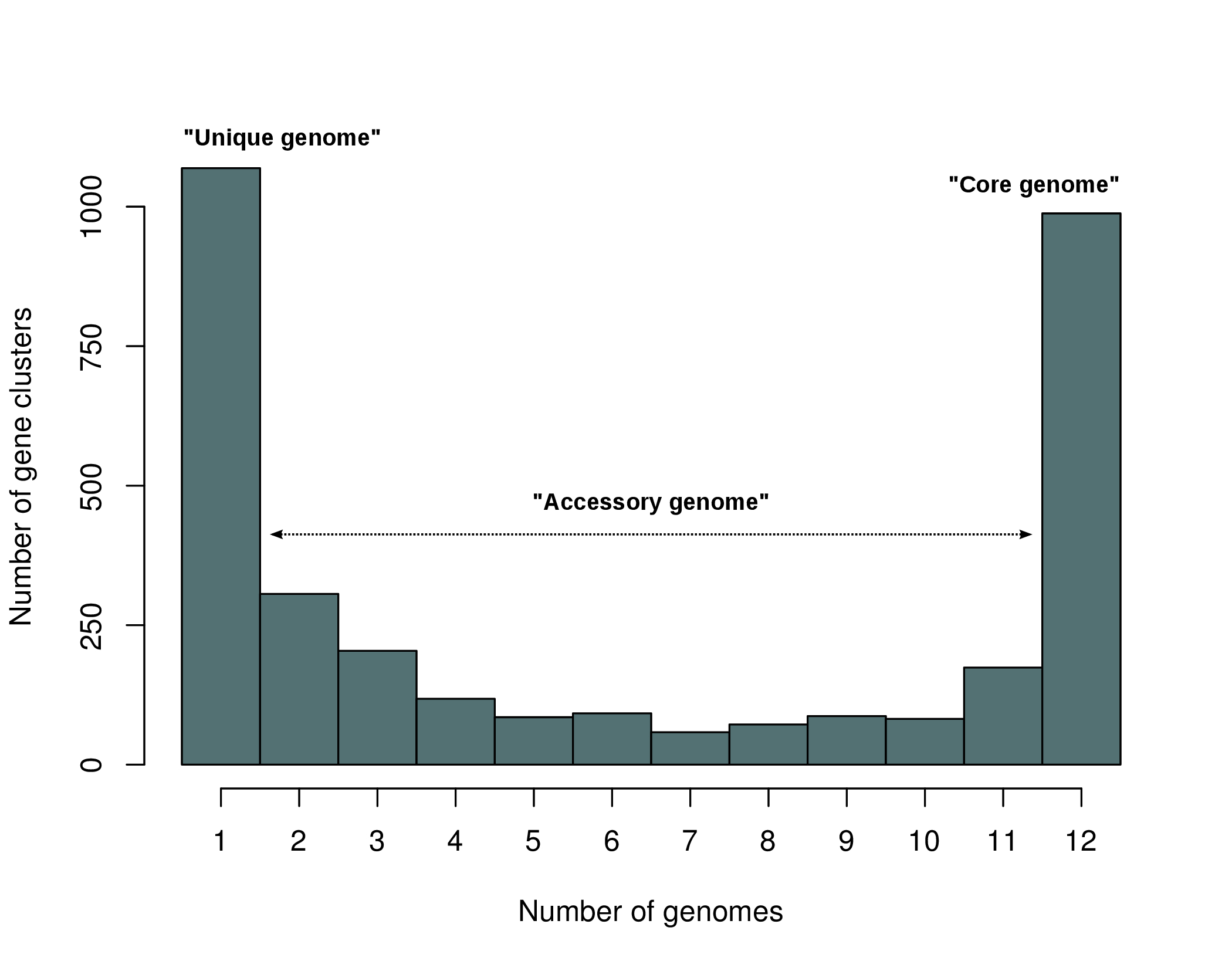

Supplement: Supplementary Figure 3 — Barplot showing the distribution of core, accessory and unique gene clusters among the 12 L. helveticus strains. The y-axis shows the number of gene clusters for every category, the x-axis shows how many strains contribute to the respective clusters. On the leftmost position (“1”) the number of clusters with gene(s) from only one strain is shown (“Unique genome”). On the rightmost position (“12”) the same is shown for the core genome (genes present in all strains). Everything in between (“2” – “11”) corresponds to gene clusters of the accessory genome. [file Image3.JPEG]

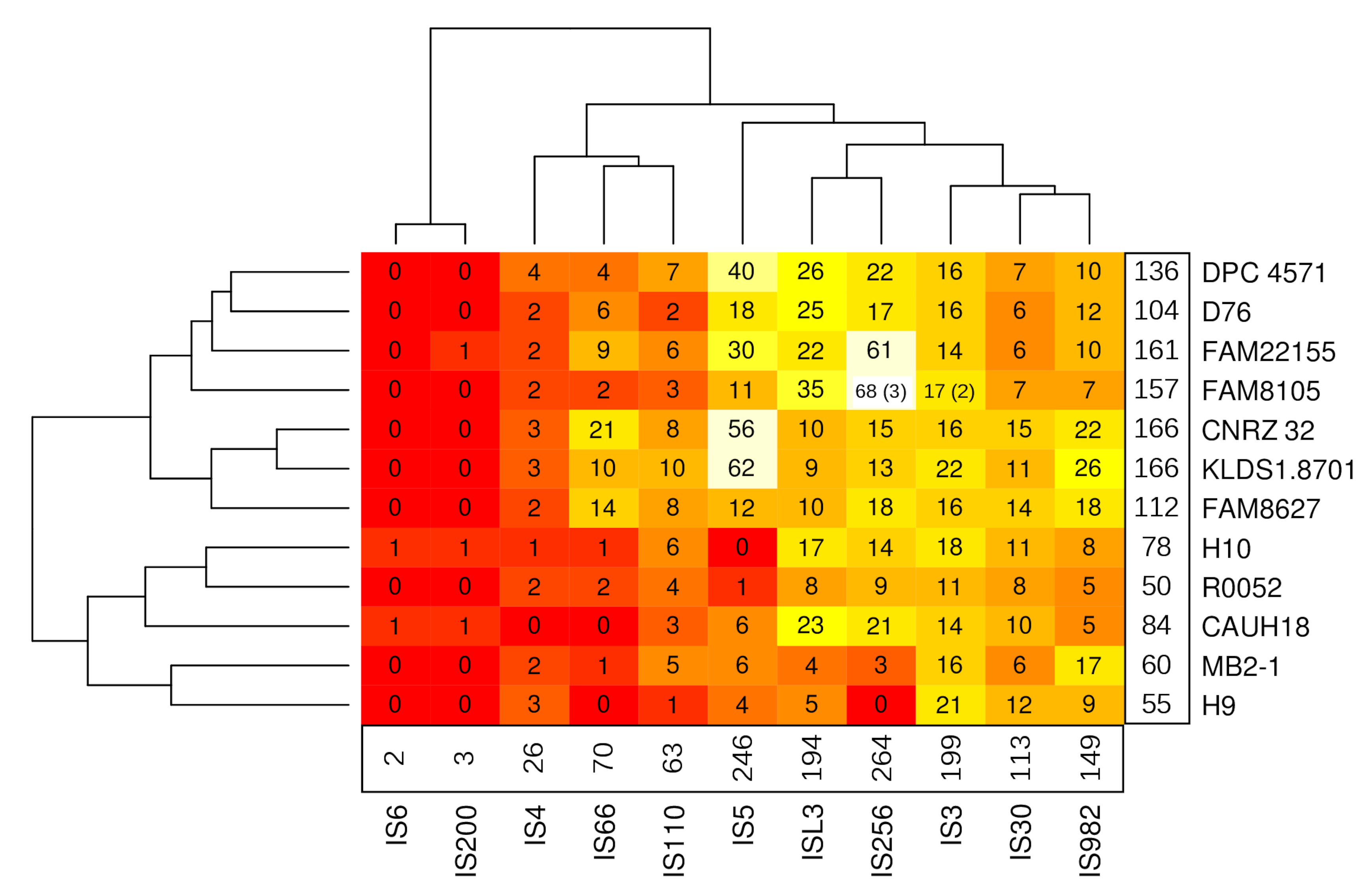

Supplement: Supplementary Figure 4 — Distribution of the occurrence of eleven insertion sequence (IS) families among 12 L. helveticus strains shown as a heatmap. The background color corresponds to the number of ISs detected using TnpPred for an IS family for the respective strain (reflecting log values used for clustering). Strains and IS are clustered (hierarchical clustering using average linkage and euclidean distance based on log values) and the dendrogram is shown on top for IS and on the left for the strains. For FAM8105, the ISs detected on the plasmid are shown in brackets. White boxes at the bottom and at the right show the total for IS families and strains, respectively. For the remaining eight families (IS1, IS1380, IS21, IS481, IS630, IS91, ISAs1, Tn3), no hits were observed. [file Image4.JPEG]
