# Supplementary material for: Comparative Genomics of Completely Sequenced Lactobacillus helveticus Genomes Provides Insights into Strain-Specific Genes and Resolves Metagenomics Data Down to the Strain Level
Source: Front Microbiol. 2018 Jan 30;9:63. doi: 10.3389/fmicb.2018.00063 (PMC5797582; doi:10.3389/fmicb.2018.00063)
Supplement: Supplementary Data Sheet 1 — Supplementary Material and Methods & Results. [file DataSheet1.DOCX]

Supplementary Material

Comparative genomics of completely sequenced *Lactobacillus helveticus* genomes provides insights into strain-specific genes and resolves metagenomics data down to the strain level

# Supplemental Material and Methods

## *In silico* analysis for gene families of interest

The protein sequences of enzymes that are involved in amino acid biosynthesis pathways (as described in the KEGG database, Feb.2015) were downloaded for the *L. helveticus* DPC 4571 strain (http://www.genome.jp/kegg-bin/show_pathway?org_name=map&mapno=01230&mapscale=1.0&show_description=hide&show_module_list=). In case this strain did not encode a particular enzyme, the sequence was downloaded from a different *Lactobacillus* reference strain. Similarly, protein sequences of selected transporters, proteinases and peptidases or other genes of interest were also analyzed. Protein sequences were then blasted (NCBI blastx) against the complete genome of the three FAM strains and five additional *Lactobacillus* genomes, respectively. A strain was listed as auxotroph for a particular amino acid when at least one enzyme from the KEGG pathway for this amino acid was absent.

## IS element analysis

HMMs for 19 IS transposase families were downloaded from the TnpPred web service (as of June 21, 2017, http://www.mobilomics.cl, (Riadi et al., 2012)). To predict IS elements, the putative CDSs of all 12 *L. helveticus* strains (including plasmids) were queried against the HMMs using HMMSCAN (HMMER 3.1b2, hmmer.org, cut-off e-value of 1e-10), and the hit with the lowest e-value was picked for the classification. The hits for the respective families were then counted for every strain, log transformed and plotted as heatmap using R (heatmap.2 from gplots package, using average linkage clustering and Euclidean distance).

On top of the TnpPred analysis of IS elements, all putative CDSs of our three FAM strains were also analyzed on the ISfinder web service (https://www-is.biotoul.fr (Siguier, 2006), as of June 21, 2017, with blastp mode and an e-value cut-off of 1e-60). The best IS element hit for every CDS was taken and the hits were counted for each group. To check for disrupted IS elements, all pseudogenes were extracted as DNA sequences and queried on ISfinder as described above but using blastn.

## Preparation of soluble and cell wall fractions

Strains were grown in 50 mL MRS broth at 37°C for 16 h. Before harvesting, OD600 was determined with a spectrophotometer (LKB Biochrom 4050 Ultrospec II) and used to normalize the CEP activity measurements. Cells were harvested by centrifugation, washed twice with Na 2 HPO 4 /NaH 2 PO 4 buffer (20 mM, pH 7,4) and resuspended in 1 mL of this buffer. Cell lysis was performed with 0.2 g glass beads (212-300 mm, Sigma-Aldrich Chemie AG, Steinheim, Germany) using an Omni Bead Ruptor (6 ms -1 for 45 s; Labforce AG, Muttenz, Switzerland). The extract was fractionated by centrifugation. The supernatant containing soluble protein was named cell-free extract (CFE). The pellet contained the cell wall fraction (CWF).

## Cell envelope protease (CEP)

CEP activity was measured using Azocasein as substrate. The cell wall fraction was resuspended in a mixture of 500 µL 2% Azocasein in 50 mM Tris-HCl (pH 7,5) and 500 µL 50 mM Imidazol in 10 mM CaCl_2_ (pH 6,5) using an Omni Bead Ruptor (6 ms^-1^ for 10 s; Labforce AG, Muttenz, Switzerland). After incubation at 37°C for 60 min the reaction was stopped by adding 500 µL 5% tri-chloric acetic acid. Uncleaved Azocasein was separated by centrifugation. 200 µL of the supernatant were transferred to a microtiter plate and the absorption was measured with a spectrophotometer (SpectraMax M2, Molecular Devices, Berkshire, UK) at 440 nm. Enzymatic activity of CEP is defined by the release of azo-dye in mol per minute and number of cells (OD600=20). Extinction coefficient: Σ_azo-dye_ = 32 M^-1^cm^-1^.

# Supplemental Results

## Insertion sequence analysis

We analyzed the 12 available complete *L. helveticus* genomes (Table 1) for the occurrence of insertion sequence (IS) elements using the two common prediction tools TnpPred (Riadi et al., 2012) and ISFinder (Siguier, 2006). This complements the analysis by Sun and colleagues (Sun et al., 2015), in our case for completely sequenced genomes, where all repeats were resolved, allowing to accurately enumerate all IS elements.

The search for IS element families using the TnpPred database in all 12 strains revealed hits for eleven of nineteen IS families in one or more of the strains (Supplementary Figure 4). The numbers ranged from 264 hits (IS256) and 246 hits (IS5) down to three hits (IS200) and two hits (IS6). Strains CNRZ 32 and KLDS1.8701 harbored most IS sequences (166), whereas R0052 had the fewest hits (50). The three strains sequenced in this study all had a relatively high count of IS elements: 157 for FAM8105, 161 for FAM22155, and 112 for FAM8627. Among the six plasmids, only that of FAM8105 harbored IS elements (three IS256, two IS3 elements) (Supplementary Figure 4). A hierarchical clustering of the strains according to their IS element distribution resulted in a topology resembling that of the phylogenetic tree. When performing the analysis using the ISfinder database (Supplementary Table 4) we did not only search CDSs against the database (blastp) but also the putative nucleotide sequences of all pseudogenes (blastn). The search with ISfinder has the advantage that for every hit the exact name of the blast hit subject can be retrieved as well as the origin/source of first isolation (Supplementary Table 4). For the CDSs, this analysis resulted in additional hits compared to TnpPred. This is mainly due to the fact that the ISfinder database harbors a broader spectrum of IS families. However, the families which are present in both TnpPred and ISfinder have a very similar number of hits found by both methods for our *L. helveticus* FAM strains.

As some of the IS elements detected by ISfinder were strain-specific, they may have potential to be developed into diagnostic tools. We also found that some IS elements were exclusively or almost exclusively present in searched pseudogenes (e.g. ISLhe11 or ISLp11).

# References

Riadi, G., Medina-Moenne, C., and Holmes, D. S. (2012). TnpPred: A Web Service for the Robust Prediction of Prokaryotic Transposases. *Comp. Funct. Genomics* 2012, 1–5. doi:10.1155/2012/678761.

Siguier, P. (2006). ISfinder: the reference centre for bacterial insertion sequences. *Nucleic Acids Res.* 34, D32–D36. doi:10.1093/nar/gkj014.

Sun, Z., Harris, H. M. B., McCann, A., Guo, C., Argimón, S., Zhang, W., et al. (2015). Expanding the biotechnology potential of lactobacilli through comparative genomics of 213 strains and associated genera. *Nat. Commun.* 6, 8322.
